# Safe reinforcement learning under temporal logic with reward design and quantum action selection

**DOI:** 10.1038/s41598-023-28582-4

**Published:** 2023-02-02

**Authors:** Mingyu Cai, Shaoping Xiao, Junchao Li, Zhen Kan

**Affiliations:** 1 Department of Mechanical Engineering, Lehigh University, 113 Research Drive, Bethlehem, PA 18015 USA; 2 Department of Mechanical Engineering, University of Iowa, 3131 Seamans Center, Iowa City, IA 52242 USA; 3 Department of Automation, University of Science and Technology of China, 443 Huangshan Road, Hefei, 230026 Anhui China

**Keywords:** Mechanical engineering, Electrical and electronic engineering

## Abstract

This paper proposes an advanced Reinforcement Learning (RL) method, incorporating reward-shaping, safety value functions, and a quantum action selection algorithm. The method is model-free and can synthesize a finite policy that maximizes the probability of satisfying a complex task. Although RL is a promising approach, it suffers from unsafe traps and sparse rewards and becomes impractical when applied to real-world problems. To improve safety during training, we introduce a concept of safety values, which results in a model-based adaptive scenario due to online updates of transition probabilities. On the other hand, a high-level complex task is usually formulated via formal languages, including Linear Temporal Logic (LTL). Another novelty of this work is using an Embedded Limit-Deterministic Generalized Büchi Automaton (E-LDGBA) to represent an LTL formula. The obtained deterministic policy can generalize the tasks over infinite and finite horizons. We design an automaton-based reward, and the theoretical analysis shows that an agent can accomplish task specifications with the maximum probability by following the optimal policy. Furthermore, a reward shaping process is developed to avoid sparse rewards and enforce the RL convergence while keeping the optimal policies invariant. In addition, inspired by quantum computing, we propose a quantum action selection algorithm to replace the existing $\varepsilon$-greedy algorithm for the balance of exploration and exploitation during learning. Simulations demonstrate how the proposed framework can achieve good performance by dramatically reducing the times to visit unsafe states while converging optimal policies.

## Introduction

Markov Decision Processes (MDPs) usually model motion planning subject to stochastic uncertainties and have been applied to represent many engineering systems^1^. On the other hand, complex high-level specifications can be expressed via formal languages^2^, including Linear and Signal Temporal logic (LTL and STL). Specifically, automaton-assisted control synthesis for general MDPs has caught growing attention to achieve tasks formulated by LTL languages. When assuming an MDP model is thoroughly knowledgeable, a common LTL-based objective is to optimize the balance of maximizing the task satisfaction probability and reducing the total expected cost^3,4^.

We are in the era of Artificial Intelligence (AI). Advanced AI techniques, including Machine Learning (ML) and Evolutionary Algorithms (EAs), have been utilized in various science and engineering disciplines. Many pioneer works have been done in materials science, complex systems, robotics, and more. As one of the ML’s subsets, Reinforcement Learning (RL) doesn’t require pre-gathered data for supervision. Indeed, RL is a sequential decision-making process, and the agent learns optimal control policies via gathering experience from the unknown environment^5^ and sometimes considering uncertain MDPs.

In RL, safety means the agent avoids visiting undesirable states in the exploration process. However, although standard RL algorithms can ensure optimal convergence, those algorithms generally lack safe protection on what happens during the learning process. For example, a mobile robot is not allowed to reach the control room, but it may visit this room using epsilon policies during learning. Consequently, the agent doesn’t intend to explore safely while learning the optimal policy to maximize the collected reward. Therefore, safe RL has attracted more attention, and various methods have been proposed^6^. However, most existing methods^7–10^ either hold strong assumptions about the dynamic models or only focus on minimizing the risk of violating a safety specification without considering complex high-level specifications.

There have been several proposed works on abstraction-based scenarios of MDPs in the past few years. For example, some research works employed LTL formulas to specify the instructions for a control agent to learn optimal strategies, i.e., optimal policies. Specifically, many works^11–15^ designed automaton-based rewards so that model-free RL agents could find the optimal policies satisfying LTL specifications with probabilistic guarantees. However, none of them addresses the critical safety issues during training.

Li et al.^16^ designed a robustness-based automaton and combined control barrier functions to facilitate learning, but the tasks introduced in^16^ were considered over finite horizons only. A related work^17^ utilized a Limit-Deterministic Generalized Büchi Automaton (LDGBA)^18^ to elucidate LTL specifications for learning enhancement. It proposed a model-based safe padding technique to prevent the system from entering bad states. However, as shown in our previous study^15^, directly applying LDGBA with purely positional policies, i.e., deterministic policies, might fail to achieve some tasks because there was no tracking record of accepting sets. An accepting set consists of automaton states that satisfy the acceptance condition of an LDGBA.

On the other hand, there is a growth of interest in quantum supremacy because a quantum computing algorithm, demonstrated on a quantum computer, offers significant speedup compared to the best possible algorithm on a classical computer. Moreover, the integration of machine learning and quantum computing^19^, called Quantum Machine Learning (QML)^20^, investigates how to encode classical data in quantum states and leverage superposition properties of quantum systems for solving some specific problems. Particularly, there is an exciting topic for researchers, Quantum Neural Networks (QNNs), including Quantum Convolutional Neural Networks (QCNNs).

Quantum neural networks are computational neural network models, mostly feed-forward networks^21^, in which quantum bits in quantum neurons process and pass the information. In addition, inspired by Convolutional Neural Networks (CNNs), QCNNs were developed and evaluated for recognizing quantum states encoded from symmetry-protected topological phases^22^. A typical application of QML was image recognition^23^ utilizing QCNN (or QNN) and Quantum Boolean Image Processing (QBIP)^24^. Furthermore, QNNs have been implemented in Deep Reinforcement Learning (DRL) to enhance learning outcomes^25^.

Quantum computing, in general, is much faster than classical computing, such as solving optimization problems^26^. Therefore, we can intuitively expect that RL and quantum computing will join forces to make faster AI. Saggio *et al.*^27^ utilized a quantum communication channel for an agent interacting with the environment to speed up the RL process. Their method generated a quantum state that was a superposition of rewarded and non-rewarded action sequences at each quantum epoch. After the environment flipped the sign of the winning action sequence, a quantum algorithm was applied to improve the chance of selecting the best action sequence. Such a hybrid AI has been shown to speed up the RL process by $60\%$. However, their approach was only applied to the Deterministic Strictly Epochal (DSE) learning scenarios.

In another work, Dong and co-workers^28^ utilized MDP states (actions) in the conventional RL as eigenbases to generate the state (action) spaces in Quantum Reinforcement Learning (QRL) by superposition. The eigenbases, i.e., eigen states or eigen actions, serve as the orthogonal bases in a Hilbert space corresponding to the generated quantum system. To update the probability of eigen actions by Grover’s algorithm, the collected reward, and state value functions are needed to determine the number of iterations. In addition, each eigen action state has a duplicated copy to prevent memory loss after selecting an action in their approach. Ganger and Hu^29^ extended Dong’s work^28^ by using state-action value functions, also named Q values, instead of state value functions in QRL.

In this paper, we propose a few advanced techniques in RL for motion planning. One of our contributions is to extend our previous results^15^ by presenting a provably correct reward design and developing the model-based safe padding. We encode LTL specifications over an infinite horizon into an Embedded LDGBA (E-LDGBA) that can record unvisited accepting sets to enable the application of deterministic policies. By using the shaping process for dense rewards, rigorous analysis shows that optimizing the expected return of the shaped reward scheme is the same as maximizing the satisfaction probability of LTL.

Secondly, assuming the ability of local observation, model-based padding can effectively add a “shield” to avoid the agent entering into sink components with a probabilistic bound and to maintain safety during the learning process. We propose a concept of safety value functions, including state and action safety values, to estimate an agent’s probability of entering a safe state. Combining RL’s conventional value functions and the proposed safety value functions will maximize the agent’s safe protection and task satisfaction.

There are some other works considering safety issues in RL. For example, Fernandez-Gauna *et al.*^30^ defined Undesirable Terminal States (UTS) as some of the terminal states associated with negative rewards. Once the agent reached one UTS, the corresponding constraints were violated, and the environment would be reset to its initial state. Another work^31^ presented an approach for provably safe learning. This work introduced Justified Speculative Control (JSC) that combined verified runtime monitoring with RL. If the system was accurately modeled, only safe actions were taken. Otherwise, any available action would be selected randomly. Differing from those works, we propose safety value functions to quantify how well the agent will avoid unsafe states starting from the current state or taking the current action. The action selection depends on the conventional RL value function and the newly-introduced safety value function.

In addition, this work improves several aspects of the result of^17^. First, we employ a novel automaton structure that has been verified to accept the same language as LDGBA, i.e., E-LDGBA, to make up for the drawbacks of LDGBA. Then, we develop a potential function in a reward-shaping process to maintain a dense reward, and the obtained optimal policy remains to satisfy the tasks with maximum probability. In addition, by dividing the LTL into two parts, one of which defines the safe properties, we show that the model-based safe padding via safety values remains the original optimal convergence invariant.

Finally, we propose a quantum action selection algorithm in this paper, inspired by Grover’s algorithm and quantum gate/measurement noises, to substitute the $\varepsilon$-greedy action selection in conventional RL. As a difference from the work^27^, the quantum state, representing the action space in our method, is created by the superposition of available actions at the current MDP state instead of action sequences at each episode^27^. On the other hand, this quantum state is locally generated at each learning step, and no duplication is needed for global updates as in the works of^28^ and^29^. It shall be noted that there is no “perfect” copy of an arbitrary unknown quantum state available, according to the no-cloning theorem^32^.

The organization of this paper is described below. Section "Problem formulation" formulates MDP, RL, LTL, automata, and the problem definition. After introducing E-LDGBA, Section "Automaton‑based reward design" describes an automaton-based reward design and a reward-shaping process. Section "Safety value functions" proposes safety value functions, and quantum action selection is described in Section "Quantum action selection". Then, two examples are included in Section "Simulations and discussions", followed by the conclusion and forthcoming works.

## Problem formulation

### Quantum computing

In classical computing, i.e., binary computing, a classical bit is a binary piece of information that can only take one of two possible values or states; for example, logic states 0 or 1. A qubit, a short name for a quantum bit, is the quantum equivalence of a classical bit. Differing from a classical bit, a qubit can represent one of two basic states or one possible combination. Given the most common basis, $\vert 0 \rangle$ and $\vert 1 \rangle$ that correspond to the logic states 0 and 1 for a classical bit, a qubit in its superposition state can be expressed as

1 $$\begin{aligned} \vert q_1 \rangle = \alpha _0 \vert 0 \rangle + \alpha _1 \vert 1 \rangle \end{aligned}$$

where $\alpha _0$ and $\alpha _1$ are complex coefficients. When we measure a qubit in its superposition state, i.e., Equation (1), the qubit would collapse into a state of the basis, e.g., $\vert 0 \rangle$ or $\vert 1 \rangle$, with the probability of $\vert \alpha _0 \vert ^2$ or $\vert \alpha _1 \vert ^2$, respectively. In addition, $\vert \alpha _0 \vert ^2 + \vert \alpha _1 \vert ^2 = 1$ shall be satisfied.

Similar to Equation (1), a quantum state in an n-qubit system can be written as

2 $$\begin{aligned} \vert \psi _n \rangle = \vert q_1 q_2 \dots q_n \rangle = \sum _{k=0}^{2^{n}-1} \alpha _k \vert k \rangle \end{aligned}$$

where $\vert k \rangle$ are basic states of the n-qubit system, and $\alpha _k$ are the corresponding complex coefficients satisfying $\sum _{k=0}^{2^{n}-1} \vert \alpha _k \vert ^2 = 1$. When being measured, the n-qubit state will collapse into one of the basic states $\vert k \rangle$ with the probability of $\vert \alpha _k \vert ^2$.

In quantum computing, a quantum register in the n-qubit system is utilized to carry a superposition of n-qubit basic states. Compared to an n-bit classical register that can store any one of $2^n$ possible numbers, an n-qubit register can store any combination of $2^n$ numbers. As the quantum version of a classical logic gate, a quantum gate operates on a quantum register that is usually initialized as $\vert 00...0 \rangle$ to evolve its state during the quantum computation.

One of the standard quantum single-qubit gates is the Hadamard gate (*H*), which results in a superposition of equal parts $\vert 0 \rangle$ and $\vert 1 \rangle$ when operating on either $\vert 0 \rangle$ or $\vert 1 \rangle$, as shown below.

3 $$\begin{aligned} H \vert 0 \rangle = \frac{1}{\sqrt{2}} \vert 0 \rangle + \frac{1}{\sqrt{2}} \vert 1 \rangle , \quad H \vert 1 \rangle = \frac{1}{\sqrt{2}} \vert 0 \rangle - \frac{1}{\sqrt{2}} \vert 1 \rangle \end{aligned}$$

Indeed, the right-hand sides of Equation (3) are another set of basic states, named $\vert + \rangle$ and $\vert - \rangle$, which are also commonly used.

In addition, among the Pauli gates (*X*, *Y*, *Z*) and the Phase gates (*S* and *T*), the *X* gate is the quantum analog of the classical *NOT* gate with respect to $\vert 0 \rangle$ and $\vert 1 \rangle$, i.e., $X \vert 0 \rangle = \vert 1 \rangle$ and $X \vert 1 \rangle = \vert 0 \rangle$. In addition, a commonly used two-qubit gate is *CNOT* or *CX* gate, which provides the quantum equivalence of the classical *XOR* gate. *CNOT* gate stands for the controlled-not gate and is always applied on two qubits. If the first qubit (as the control one) is $\vert 1 \rangle$, then a *NOT* operation is applied to the second qubit (as the target one). Otherwise, the target qubit remains the same. For example,

4 $$\begin{aligned} CNOT \vert 01 \rangle = \vert 01 \rangle , \quad CNOT \vert 11 \rangle = \vert 10 \rangle \end{aligned}$$

### Markov decision process

Mathematically, an MDP, denoted by ${\mathcal {M}}=\left( S,A,p_{S}, s_{0},\Pi ,L\right)$, consists of a finite state space *S* and a finite action space *A*. In addition, *A*(*s*) at state *s* denotes the available actions the agent can take at this state. The transition function, $p_{S}:S\times A\times S \rightarrow [0,1]$, defines the probability of the agent moving from one state (*s*) to another ($s'$) after it takes an action (*a*). Particularly, $\sum _{s'} p_S(s,a,s')=1$ shall be satisfied. Usually, the transition probability $p_{S}$ describes the motion uncertainties in an RL problem. $\Pi$ is a set of atomic propositions. The labeling function, $L:S \rightarrow 2^{\Pi }$ where $2^{\Pi }$ is the power set of $\Pi$, assigns a subset of $\Pi$ to each state. Sometimes, there exists an initial state $s_{0}\in S$ with which the agent starts.

When an action function, $\xi :S\rightarrow A$, is deterministic, it outputs an action (*a*) at a given state (*s*). The MDP evolves gradually after performing action $\xi _{i} = a$ at step *i* ($i \ge 0$). Consequently, a sequence of actions $\varvec{\xi }=\xi _{0}\xi _{1}\ldots$ defines the control policy and generates a path ${\varvec{s}}=s_{0}s_{1}s_{2}\ldots$ over ${\mathcal {M}}$. It shall be noted that the transition probability $p_{S}\left( s_{i},a_{i},s_{i+1}\right)$ is positive for all *i*. For a stationary policy, we have $\xi _{i}=\xi$ for all *i*. On the other hand, if $\xi _i =\xi (s_i)$ depends on the current state $s_i$ only, the control policy is memoryless. Otherwise, the policy shall have a finite memory with the history of visited states, i.e., $\xi _i = \xi (..., s_{i-1}, s_i)$.

An MDP has a Markov property if the environment is fully observable and an agent carries out a simple go-to-goal task. Consequently, each decision-making relies on the current state only, so the control policy is memoryless. However, one of this study’s focuses is developing a framework to handle complex tasks by introducing a product of MDP and LTL-induced automaton. The induced control policy is usually a finite memory one, i.e., the action selection depends on the current and past states.

### Reinforcement learning

**Figure 1:**
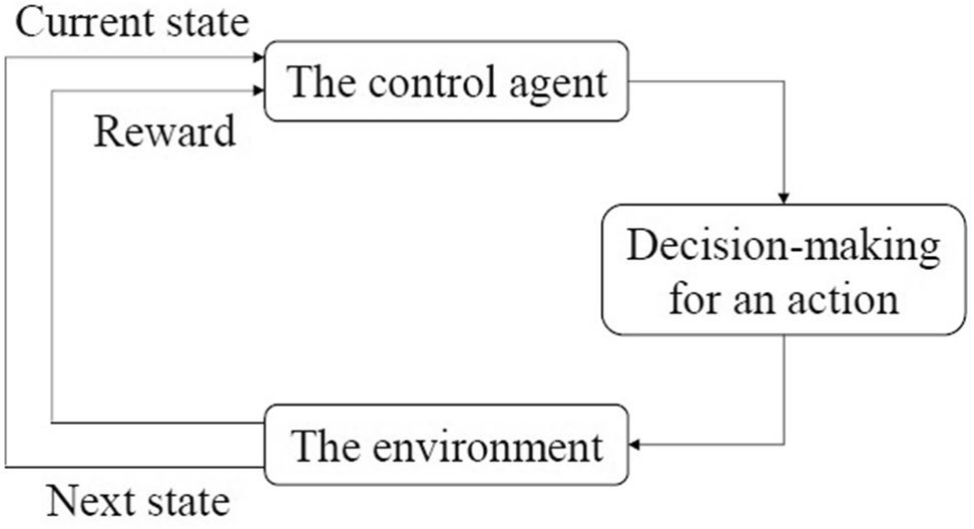
The agent interacts with the environment in a reinforcement learning problem.

The environment in an RL problem can be expressed by an MDP as defined in Section "Markov decision process". An agent learns the policy via communicating with the environment, and learning is an iterative process. For example, Fig. 1 shows that the agent decides which action to take after identifying the present state. Once conducting the selected action, it receives feedback, i.e., the reward, and observes the next state for decision-making of the following action.

The reward function is one of the key elements in an RL problem. Generally, a reward function can be denoted by $\varLambda (s, a, s')$, which generates the reward to an agent after it takes an action (*a*) at the current state (*s*) and reaches another state ($s'$). In addition, a discount function $\gamma (s, a, s') \in [0,1]$ is usually employed, and here defines the expected discounted return an agent can receive under policy $\varvec{\xi }$ after starting from state *s*.

5 $$\begin{aligned} U^{\varvec{\xi }}\left( s\right) ={\mathbb {E}}^{\varvec{\xi }}\left[ \sum _{i=0}^{\infty }\gamma ^{i} \left( s_{i},a_{i},s_{i+1} \right) \cdot \varLambda \left( s_{i},a_{i},s_{i+1}\right) \Big \vert s_{0}=s \right] \end{aligned}$$

Equation (5) is also implied as the utility or the state value function generated from policy $\varvec{\xi }$. In an RL problem, the state value function *U*(*s*) quantifies how well the agent can reach a goal state over the long run, starting from state *s*. The learning objective is finding an optimal policy, $\varvec{\xi }^{*}={\textrm{argmax}}_{\varvec{\xi }}U^{\varvec{\xi }}\left( s\right)$, which can maximize the state value at each state. On the other hand, an optimal policy guides the agent in deciding on the best action to accomplish the task. When the environment is not fully known, i.e., the transition probability in its MDP model is unknown, model-free RL methods with tabular approaches are usually utilized for finite state and action spaces.

The methods in RL can be categorized as policy-based or value-based. The value-based RL methods directly solve and converge the value functions as the optimal ones. Q-learning^33^ is one of them, and it solves action values or Q values instead of state values. A Q value is a function of state and action, i.e., *Q*(*s*, *a*), represents the expected return an agent can collect under the current policy after taking action *a* at state *s*. Once the optimal action values are converged, we can obtain an optimal policy via the greedy action selection. It shall be noted that state values are related to action values by $U(s) = \max _a Q(s,a)$.

Since Q-learning doesn’t require a state-to-state transition function, it is one of the model-free RL methods. When an RL problem has finite state/action spaces, a Q table is adopted in the naïve Q-learning method to store the value of every action at each state. Therefore, the best action can be determined at each state by searching for the highest Q value in the table. This method employs Monte Carlo simulations to converge Q values. The learning usually takes many episodes. Each episode consists of many steps or ends once the agent accomplishes the task. At each step, after taking an action (*a*), the agent moves from one state (*s*) to another ($s'$). Then, the Q value of action *a* at the current state *s* can be updated via the Bellman equation^5^.

6 $$\begin{aligned} Q_{\text {new}}(s,a) = Q(s,a)+\alpha \left[ \varLambda (s,a,s')+\gamma (s,a,s') \max _{a'} Q(s',a')-Q(s,a) \right] \end{aligned}$$

where $\max _{a'} Q(s',a')$ represents the highest action value at state $s'$.

Equations (5) and (6) include generalized definitions of the reward function $\varLambda (s,a,s')$ and the discount factor $\gamma (s,a,s')$. Indeed, in a commonly used formulation^5,33^, they are functions of the current state only, as $\varLambda (s)$ and $\gamma (s)$, respectively. In addition, a properly designed learning rate, $\alpha$, is essential. If $\alpha$ is large, the convergence is fast but sometimes unstable. In addition, non-optimal value functions may be reached. On the other hand, although a smaller $\alpha$ can result in a smoother and more stable convergence procedure, the procedure may be slower. It is practical to employ an adaptive learning rate scheme to start with a large learning rate and decrease it with iterations.

Deep neural networks are usually employed to approximate value functions if the state and/or action spaces in an RL problem are large or continuous. The methods are then called Deep Reinforcement Learning (DRL) methods. The commonly used methods include Deep Q Network (DQN)^34^, a variation of Q-learning, and Proximal Policy Optimization (PPO)^35^, a policy-based method.

### Linear temporal logic

Instead of simple go-to-goal tasks, this paper considers complex high-level tasks that LTL, a formal language, can describe. Specifically, we can identify and verify properties about how a world changes over time using LTL. The properties of interest, given high-level specifications of a system, include safety (i.e., anything terrible will never happen), liveness (i.e., something good will finally occur), and fairness (i.e., independent subsystems can make progress).

An LTL formula can be built on atomic propositions $\Pi$, some Boolean operators, including $\text {True}$, negation $\lnot$, and conjunction $\wedge$, and two temporal operators such as next $\bigcirc$ and until ${\mathcal {U}}$^2^. Its syntax can be inductively defined as

7 $$\begin{aligned} \phi := \text {True} \mid a \mid \phi _1 \wedge \phi _2 \mid \lnot \phi \mid \bigcirc \phi \mid \phi _1 {\mathcal {U}} \phi _2\,, \end{aligned}$$

where $a\in \Pi$ is an atomic proposition. The temporal operators define time-dependent properties when the system evolves. For example, the formula $\bigcirc \phi$ can be read as “$\phi$ is true at the next state” while $\phi _1 {\mathcal {U}} \phi _2$ as “$\phi _1$ is true at each state until $\phi _2$ is true at some future states.”

A word is defined as an infinite sequence ${\varvec{o}}=o_{0}o_{1}\ldots$ with $o_{i}\in 2^{\Pi }$. Here denotes $\models$ the satisfaction relation. Consequently, words can be used to interpret the LTL formula’s semantics, defined as below.

$$\begin{aligned} \begin{array}{lcl} {\varvec{o}} \models \text {True} \\ {\varvec{o}} \models \alpha &{} \Leftrightarrow &{} \alpha \in L({\varvec{o}}[0]) \\ {\varvec{o}} \models \phi _{1}\wedge \phi _{2} &{} \Leftrightarrow &{} {\varvec{o}} \models \phi _{1} \text { and } {\varvec{o}} \models \phi _{2} \\ {\varvec{o}} \models \lnot \phi &{} \Leftrightarrow &{} {\varvec{o}} \mid \ne \phi \\ {\varvec{o}} \models \bigcirc \phi &{} \Leftrightarrow &{} {\varvec{o}}[1:] \models \phi \\ {\varvec{o}} \models \phi _1 {\mathcal {U}} \phi _2 &{} \Leftrightarrow &{} \exists t_1 \text { s.t. }{\varvec{o}}[t_1:]\models \phi _{2}, \forall t_2\in [0,t_1), {\varvec{o}}[t_2:]\models \phi _{1} \\ \end{array} \end{aligned}$$

In addition to the standard operators introduced in Equation (7), we can derive other propositional and temporal logic operators, including $\text {False} \lnot$, disjunction $\vee$, implication $\rightarrow$, always $\square$, and eventually $\lozenge$^2^. It shall be noted that the basic formulas of other temporal operators, $\square \phi$ and $\lozenge \phi$, can be read as “$\phi$ is always true in the future” and “$\phi$ could be true sometimes in the future,” respectively. Thus, in an RL problem, an LTL formula can describe whether or not a set of infinite traces (i.e., sequences of MDP states) can satisfy the user-specified task(s).

**Figure 2:**
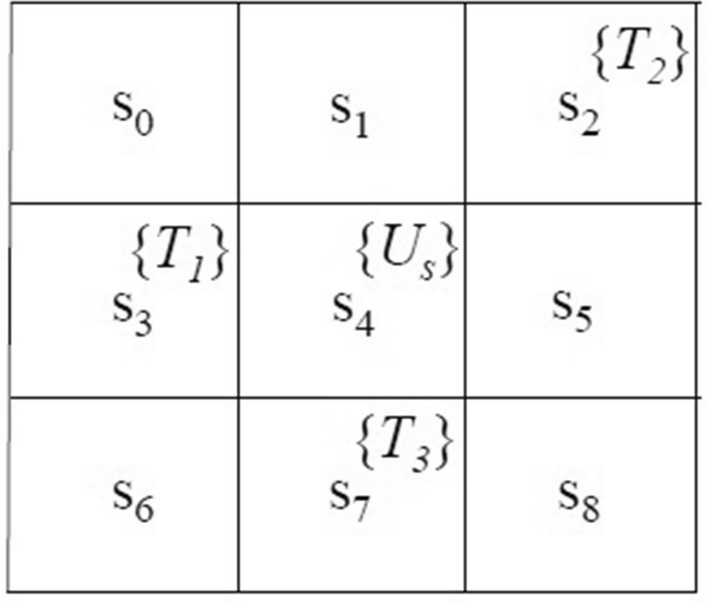
A grid-world with states of interest labeled by $T_i$ and unsafe states labeled by $U_s$.

#### Example 1

Given a grid-world, as shown in Fig. 2, an MDP ${\mathcal {M}}$ can be defined with $s=\{s_0, s_1, ..., s_8\}$ and $\Pi =\{T_1, T_2, T_3, U_s\}$. $T_i$ where $i\in {1,2,3}$ are labeled on the states of interest while $U_s$ is labeled on an unsafe state. Considering a user-specified task, “First $T_1$, then $T_2$, finally $T_3$, and never $U_s$,” we can write an LTL formula as $\varphi =\lozenge \left( T_1\wedge \lozenge \mathtt { \left( T_2 \wedge \lozenge \mathtt {T_3} \right) } \right) \wedge \lnot U_s$. This task can be satisfied by, for example, a word of ${\varvec{o}}= T_1 {T_1}^* T_2 T_3 {T_3}^*$ where $*$ matches the preceding character for 0 or more times (up to infinite). Consequently, a set of infinite traces will satisfy the task defined in this example if it can generate the above-expressed word.

In the above example, the solution consists of any policy that can generate traces to satisfy the LTL-formulated task(s) as well as maximize the expected return in Equation (5). In other words, the LTL formula plays a constraint in finding the optimal policy. However, such a constraint cannot be directly implemented in conventional RL problems. Instead, a finite state automaton is usually employed to represent the LTL formula. Then, the optimal policy can be achieved via RL with automaton theory and model checking^2^.

### Limit-deterministic generalized Büchi automaton

As discussed above, an LTL formula can specify a complex high-level task. Then, task satisfaction can be evaluated by an automaton, such as an LDGBA^18^, through model checking.

#### Definition 1

*(LDGBA)* An LDGBA, ${\mathcal {A}}=\left( Q,\Sigma ,\delta ,q_{0},F\right)$, consists of a finite set of states *Q* and a finite alphabet $\Sigma =2^{\Pi }$ with a set of atomic propositions $\Pi$. The transition function, $\delta (q, \alpha ):$ $Q\times \left( \Sigma \cup \left\{ \epsilon \right\} \right) \rightarrow 2^{Q}$, allows the LDGBA to change its state when taking an input symbol ($\alpha \in \Sigma$) or not ($\alpha \in \{\epsilon \})$. In addition, $q_{0}\in Q$ is an initial automaton state. $F=\left\{ F_{1},F_{2},\ldots ,F_{f}\right\}$ represents a set of accepting sets in the automaton, and $F_{i}\subseteq Q$ where $\forall i = 1,\ldots f$. The state set *Q* in an LDGBA can be divided into deterministic and non-deterministic sets, i.e., $Q_{D}$ and $Q_{N}$, respectively. Such partition satisfies the following:

- The LDGBA transitions in the deterministic set are total, i.e., $\vert \delta \left( q,\alpha \right) \vert =1$ with $\alpha \in \Sigma$. Such transitions are allowed within this set only. Therefore, after consuming an input symbol $\alpha$ at an automaton state *q*, the resulted automaton state is $\delta \left( q,\alpha \right) \subseteq Q_{D}$,
- The state transitions without any input symbol, i.e., $\epsilon$-transitions, are only valid for state transitions from the non-deterministic set ($Q_{N}$) to the deterministic set ($Q_{D}$). Therefore, they are not allowed in $Q_{D}$. and
- All the accepting sets are subsets of the deterministic set.

A run of an LDGBA can be written as ${\varvec{q}}=q_{0}q_{1}\ldots$. Let $\inf \left( {\varvec{q}}\right)$ denotes the infinite portion of ${\varvec{q}}$. If there exists $\inf \left( {\varvec{q}}\right) \cap F_{i}\ne \emptyset$, $\forall i\in \left\{ 1,\ldots f\right\}$, we say that ${\varvec{q}}$ satisfies the LDGBA acceptance condition or the LDGBA accepts ${\varvec{q}}$. Example 2 demonstrates an LDGBA, representing the LTL formula in Example 1. We recommend Owl^36^ to readers for more details about automaton generation.

#### Example 2

**Figure 3:**
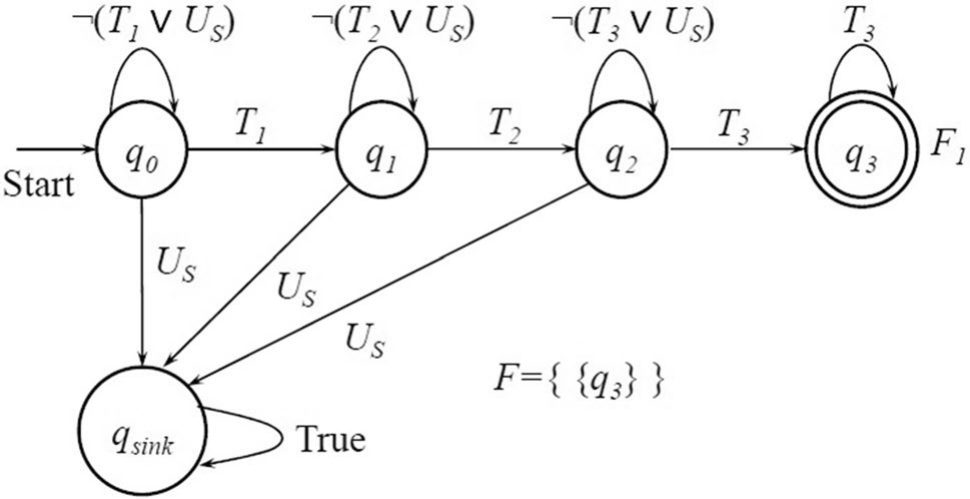
An LDGBA for the LTL formula $\phi =\lozenge \left( T_1\wedge \lozenge \mathtt { \left( T_2 \wedge \lozenge \mathtt {T_3} \right) } \right) \wedge \lnot U_s$.

In Example 1, the LTL formula is expressed as $\phi =\lozenge \left( T_1\wedge \lozenge \mathtt { \left( T_2 \wedge \lozenge \mathtt {T_3} \right) } \right) \wedge \lnot U_s$ for the user-specified task. Figure 3 shows the LTL-induced LDGBA, which has only one accepting set $F_1=\{ q_3\}$. A run of LDGBA, for example, ${\varvec{q}}=q_{0}q_{1} q_2 q_3$, is accepted by this LDGBA because ${\varvec{q}} \cap F_1 = \{q_3\}$.

### Problem formulation

It was discussed above that an LTL formula $\phi$ can describe the task specifications to be performed by an agent. For example, given a policy $\varvec{\xi }=\xi _{0}\xi _{1}\ldots$ that the agent learns from MDP ${\mathcal {M}}$, it can generate a path, denoted by ${\varvec{s}}_{\infty }^{\varvec{\xi }}=s_{0}\ldots s_{i}s_{i+1}\ldots$, with $p_S(s_i, a_i, s_{i+1})>0$. Correspondingly, a sequence of labels (i.e., a trace) can be derived from the labeling function as $L\left( {\varvec{s}}_{\infty }^{\varvec{\xi }}\right) =l_{0}l_{1}\ldots$ where $l_{i}\in L\left( s_{i}\right)$. If this trace satisfies the task, i.e., $L\left( {\varvec{s}}_\infty ^{\varvec{\xi }}\right) \models \phi$, the satisfaction probability that the agent can accomplish the task is expressed as

8 $$\begin{aligned} \Pr {}_{{\mathcal {M}}}^{\varvec{\xi }}\left( \phi \right) =\Pr {}_{{\mathcal {M}}}^{\varvec{\xi }} \left( L \left( {\varvec{s}}_{\infty }^{\varvec{\xi }} \right) \models \phi \big \vert {\varvec{s}}_{\infty }^{\varvec{\xi }} \in {\varvec{S}}_{\infty }^{\varvec{\xi }} \right) \end{aligned}$$

where ${\varvec{S}}_{\infty }^{\varvec{\xi }}$ is defined as a set of admissible paths generated from policy ${\varvec{\xi }}$, starting with the initial state $s_{0}$.

#### Assumption 1

At least one deterministic policy exists such that the agent can accomplish the task with a non-zero probability by following this policy.

Assumption 1 states that the agent can always find a policy from which an induced trace can fully satisfy the user-specified LTL task. This assumption is moderate and has been widely utilized^11,14,37^. This paper considers the LTL task as the form $\phi =\phi _{g}\wedge \square \phi _{safe}$, where $\phi _{g}$ provides a general form of high-level tasks, and $\phi _{safe}$ represents the safety requirement. Consequently, we define the problem as follows.

#### Problem 1

This RL problem considers (1) a user-specified task that can be formulated via LTL as $\phi =\phi _{g}\wedge \square \phi _{safe}$ and (2) an MDP ${\mathcal {M}}$ in which the transition function is unknown. The objective is to find a deterministic policy $\varvec{\xi ^{*}}$, which can maximize the probability of task satisfaction, i.e., $\varvec{\xi ^{*}}={\textrm{argmax}}_{\varvec{\xi }}\Pr {}_{{\mathcal {M}}}^{\varvec{\xi }}\left( \phi _g\right)$, and maintain the safety $\phi _{safe}$ during the learning process.

To address Problem 1, Section "Automaton‑based reward design" proposes an automaton-based reward design to guide the agent in learning the optimal policy on the product of MDP and automaton. Then, Section "Safety value functions" develops a safe padding technique by introducing safety value functions to promote safety during the learning process. In addition, a quantum action selection technique is proposed in Section "Quantum action selection" to substitute the $\varepsilon$-greedy algorithm for the balance of exploration and exploitation in learning.

## Automaton-based reward design

Previous work^15^ has shown that directly utilizing LDGBA may fail to find deterministic policies satisfying LTL specifications. This issue can be addressed by designing an E-LDGBA.

### Embedded LDGBA

To keep tracking unvisited accepting sets in an LDGBA, we introduce a tracking-frontier set *T* during model checking. In addition, a Boolean variable, ${\mathcal {B}}$, is employed to indicate the satisfaction of accepting conditions for each round. Here, one round is defined as all accepting sets being visited. On the other hand, this tracking-frontier set is initialized as $T=F$ while ${\mathcal {B}}$ as False. Then, the set $\left( T,{\mathcal {B}}\right) =f_{V}\left( q,T\right)$ is updated synchronously during learning process as:

9 $$\begin{aligned} f_{V}\left( q,T\right) =\left\{ \begin{array}{cc} \left( T\setminus F_{j},{\text {False}}\right) , &{} \text {if }q\in F_{j}\text { and }F_{j}\in T,\\ \left( F\setminus F_{j},{\text {True}}\right) &{} \text {if }{q\in F_{j}\text { and }T=\emptyset },\\ \left( T,{\text {False}}\right) , &{} \text {otherwise. } \end{array}\right. \end{aligned}$$

#### Definition 2

*(E-LDGBA)* Considering an LDGBA ${\mathcal {A}}=\left( Q,\Sigma ,\delta ,q_{0},F \right)$ and a Boolean variable ${\mathcal {B}}$, we can define an E-LDGBA correspondingly as $\mathcal {\overline{A}}=\left( \overline{Q},\Sigma ,\overline{\delta },\overline{q}_{0},\overline{F},f_{V},T, {\mathcal {B}}\right)$ with initially setting $T=F$ and ${\mathcal {B}}=$False, respectively. The automaton states are augmented, i.e., $\overline{Q}=Q\times 2^{F}$ or $\overline{q}=(q,T)$. In the transition function, $\overline{\delta }:\overline{Q}\times \left( \Sigma \cup \left\{ \epsilon \right\} \right) \rightarrow 2^{\overline{Q}}$, the finite alphabet $\Sigma$ is the same as in LDGBA. In addition, the non-deterministic set $\overline{Q}_N$, the deterministic set $\overline{Q}_D$, and $\epsilon$-transitions from $\overline{Q}_N$ to $\overline{Q}_D$ can be correspondingly constructed. Similarly, the set of accepting sets in E-LDGBA is $\overline{F}=\left\{ \overline{F}_{1},\overline{F}_{2}\ldots \overline{F}_{f}\right\}$, where $\overline{F}_{j}=\left\{ \left( q, T\right) \in \overline{Q}\vert q\in F_{j}\wedge F_{j} \subseteq T\right\}$, $j=1,\ldots f$. In particular, the transition $\overline{q}'=\overline{\delta }\left( \overline{q},\overline{\sigma }\right)$, where $\overline{\sigma } \in \left( \Sigma \cup \left\{ \epsilon \right\} \right)$, shall satisfy the following: 1) the transitions in LDGBA are valid, i.e., $q'=\delta \left( q,\overline{\sigma }\right)$, and 2) *T* and ${\mathcal {B}}$ are synchronously updated via $\left( T,{\mathcal {B}}\right) =f_{V}\left( q',T\right)$ at each transition as defined in Eq. (9).

We abuse the tuple structure in Definition 2 because the tacking-frontier set *T* and the Boolean variable ${\mathcal {B}}$ are synchronously updated after each transition. While updating the frontier set *T*, the Boolean variable ${\mathcal {B}}$ indicates whether the current state belongs to at least one accepting set that has not been visited within the current round. This design is essential to guide the agent to visit all accepting sets once per round with infinite rounds based on user-specified tasks.

In the rest of this paper, given an LTL formula $\phi$, we utilize ${\mathcal {A}}_{\phi }$ and $\mathcal {\overline{A}}_{\phi }$ to represent an LDGBA and its corresponding E-LDGBA, respectively. Here assume that ${\mathcal {L}}({\mathcal {A}}_{\phi })\subseteq \Sigma ^{\omega }$ is the language accepted by ${\mathcal {A}}_{\phi }$. In other words, this language is the set of all infinite words that satisfy the LTL formula $\phi$^2^. If ${\mathcal {L}}(\mathcal {\overline{A}}_{\phi })\subseteq \Sigma ^{\omega }$ is the language accepted by $\mathcal {\overline{A}}_{\phi }$, we have the following lemma.

#### Lemma 1

Given an LTL formula $\phi$, we can generate two automata: an LDGBA ${\mathcal {A}}_{\phi }=\left( Q,\Sigma ,\delta ,q_{0},F\right)$ and its corresponding E-LDGBA $\mathcal {\overline{A}}_{\phi }=\left( \overline{Q},\Sigma ,\overline{\delta },\overline{q}_{0},\overline{F},f_{V},T, {\mathcal {B}}\right)$. Then, it leads that

10 $$\begin{aligned} {\mathcal {L}}(\mathcal {\overline{A}}_{\phi })={\mathcal {L}}({\mathcal {A}}_{\phi }). \end{aligned}$$

The proof of Lemma 1 is in our previous work^15^. Therefore, we can employ E-LDGBA to uphold task satisfaction. In addition, we define a set of sink components in an E-LDGBA as below.

#### Definition 3

A set of non-accepting sink components in an E-LDGBA, i.e., $\overline{Q}_{sink}\subseteq \overline{Q}$, is a group of automaton states from which none of the accepting states can be reached.

In addition, after defining the LTL-formulated task as $\phi =\phi _{g} \wedge \square \phi _{safe}$, we can always find a set of states $\overline{Q}_{unsafe}\subseteq \overline{Q}_{sink}$ associated with the violation of $\phi _{safe}$.

### Embedded product MDP (EP-MDP)

#### Definition 4

*(EP-MDP)* Considering an MDP ${\mathcal {M}}=\left( S,A,p_{S}, s_{0},\Pi ,L\right)$ and an LTL-converted E-LDGBA $\mathcal {\overline{A}_\phi }=\left( \overline{Q},\Sigma ,\overline{\delta },\overline{q}_{0},\overline{F},f_{V},T, {\mathcal {B}}\right)$, we can construct an embedded product MDP (EP-MDP), ${\mathcal {P}}={\mathcal {M}}\times \mathcal {\overline{A}}_{\phi }=\left( X,U^{{\mathcal {P}}},p^{{\mathcal {P}}},x_{0},F^{{\mathcal {P}}}, f_{V},T, {\mathcal {B}}\right)$, which consists of a set of labeled states $X=S\times 2^{\Pi }\times \overline{Q}$ with $x=\left( s,l,\overline{q}\right) = \left( s,l,q,T\right) \in X$ and $l\in L\left( s\right)$ and a set of actions $U^{{\mathcal {P}}}=A\cup \left\{ \epsilon \right\}$. Corresponding to the restriction in the E-LDGBA, $\epsilon$-transitions are valid from $x=(s,l,\overline{q})$ with $\overline{q} \in \overline{Q}_{N}$ to $x'=(s,l,\overline{q}')$ with $\overline{q}' \in \overline{Q}_{D}$ only. The transition probability $p^{{\mathcal {P}}}\left( x,u^{{\mathcal {P}}},x'\right) :X\times U^{{\mathcal {P}}}\times X\rightarrow [0,1]$ equals (1) $p_{S}\left( s,a,s^{\prime }\right)$ if $\overline{\delta }\left( \overline{q},l\right) =\overline{q}^{\prime }$ and $u^{{\mathcal {P}}}=a\in A\left( s\right)$; (2) 1 if ${u^{{\mathcal {P}}}\in \left\{ \epsilon \right\} }$, $\overline{q}'\in \overline{\delta }\left( \overline{q},\epsilon \right)$, and $\left( s',l'\right) =\left( s,l\right)$; and (3) 0 otherwise. In addition, $x_{0}=\left( s_{0},l_{0},\overline{q}_{0}\right)$ is the initial state, and $F^{{\mathcal {P}}}=\left\{ F_{1}^{{\mathcal {P}}},F_{2}^{{\mathcal {P}}}\ldots F_{f}^{{\mathcal {P}}}\right\}$ is the set of accepting sets where $F_{j}^{{\mathcal {P}}}=\left\{ \left( s,l,\overline{q}\right) \in X \Big \vert \overline{q}\in \overline{F}_{j}\right\}$, $j=1,\ldots f$. After each transition is completed, *T* and ${\mathcal {B}}$ are synchronously updated via $\left( T,{\mathcal {B}}\right) =f_{V}\left( q',T\right)$ in Equation (9).

It shall be noted that the EP-MDP ${\mathcal {P}}$ can capture the dynamical interchanges between all possible paths over the associated MDP and all words recognized by the corresponding E-LDGBA. Assuming $\varvec{\pi }$ is a policy over ${\mathcal {P}}$ and generates an infinite path, e.g., ${\varvec{x}}_{\infty }^{\varvec{\pi }}=x_{0}\ldots x_{i}x_{i+1}\ldots$. This path is acceptable if $\inf \left( {\varvec{x}}_{\infty }^{\varvec{\pi }}\right) \cap F_{i}^{{\mathcal {P}}}\ne \emptyset$ where $i=1,\ldots f$. Such an accepting path ${\varvec{x}}_{\infty }^{\pi }$ can produce a policy $\varvec{\xi }$ over ${\mathcal {M}}$ to satisfy the LTL-specified task $\phi$. On the other hand, ${\mathcal {P}}$ has at least one accepting maximum end component (AMEC)^2^, ${\mathcal {P}}'_{\left( X',U'\right) }$, and reaching this AMEC is the same as satisfying task $\phi$.

As mentioned above, this study decomposes an LTL-formulated task into $\phi =\phi _{g}\wedge \square \phi _{safe}$, and we can define a set of unsafe states as $X_{unsafe}=\left\{ x=(s,l,\overline{q})\in X \vert s\in S\wedge \overline{q}\in \overline{Q}_{unsafe}\right\}$. Also, let $\Pr ^{\mathbf {\varvec{\pi }}}\left[ x\models \text {Acc}_{{\mathcal {P}}}\right]$ denote the probability of policy $\varvec{\pi }$ satisfying the EP-MDP’s acceptance conditions. Then, the maximum probability of satisfying the acceptance of ${\mathcal {P}}$ is defined as $\Pr _{max}\left[ x\models \text {Acc}_{{\mathcal {P}}}\right] ={\max _{\varvec{\pi }}}\Pr _{{\mathcal {M}}}^{\varvec{\pi }}\left( \text {Acc}_{{\mathcal {P}}}\right)$. Consequently, Problem 1 can be rephrased as below.

#### Problem 2

Considering an MDP ${\mathcal {M}}$ and an LTL task $\phi$, a corresponding EP-MDP ${\mathcal {P}}$ exists, and transition probabilities are unknown. The objective is to find a policy $\varvec{\pi }^{*}$ that satisfies the EP-MDP’s acceptance conditions with a maximum probability, i.e., $\Pr ^{\varvec{\pi }^{*}}\left[ x\models \text {Acc}_{{\mathcal {P}}}\right] =\Pr _{max}\left[ x\models \text {Acc}_{{\mathcal {P}}}\right]$, and avoid entering $X_{unsafe}$ during the learning process.

A base reward design will be discussed in the following subsection. Such a design can enable the RL agent to find the optimal policy and achieve the maximum probability of satisfying task(s). Then, we will further improve the reward density via reward shaping with a potential function.

### Base reward

In an EP-MDP ${\mathcal {P}}$, all accepting states can be grouped into a set as $F_{U}^{{\mathcal {P}}}=\left\{ x\in X \vert x\in F_{i}^{{\mathcal {P}}},\forall i\in \left\{ 1,\ldots f\right\} \right\}$. According to the reward function $\Lambda (s, a, s')$ defined in an MDP ${\mathcal {M}}$, after each transition $\left( x,u^{{\mathcal {P}}},x'\right)$ in the corresponding EP-MDP, the agent can receive a reward as: 1) $R\left( x,u^{{\mathcal {P}}},x'\right) =\Lambda \left( s,a,s^{\prime }\right)$ if $\overline{\delta }\left( \overline{q},l\right) =\overline{q}^{\prime }$ and $u^{{\mathcal {P}}}=a\in A\left( s\right)$; and 2) $R\left( x,u^{{\mathcal {P}}},x'\right) =0$ otherwise. In addition, the discount factor in the EP-MDP can be defined similarly. In this study, we consider both functions depending on the state only. Consequently, inspired by^14^, the reward and discount factor functions can be designed as below.

11 $$\begin{aligned} R\left( x\right)= & {} \left\{ \begin{array}{cc} 1-r_{F}, &{} \text {if }x\in F_{U}^{\mathcal {{\mathcal {P}}}},\\ 0, &{} \text {otherwise,} \end{array}\right. \end{aligned}$$

12 $$\begin{aligned} \gamma \left( x\right)= & {} \left\{ \begin{array}{cc} r_{F}, &{} \text {if }x\in F_{U}^{\mathcal {{\mathcal {P}}}},\\ \gamma _{F}, &{} \text {otherwise,} \end{array} \right. \end{aligned}$$

where $\gamma _F$ is the discount factor when the agent doesn’t reach any accepting state after a valid transition, and $r_{F}\left( \gamma _{F}\right)$ otherwise. In addition, $r_{F} (\gamma _F)$ shall satisfy $\underset{\gamma _{F}\rightarrow 1^{-}}{\lim }r_{F}\left( \gamma _{F}\right) =1$ and $\underset{\gamma _{F}\rightarrow 1^{-}}{\lim }\frac{1-\gamma _{F}}{1-r_{F}\left( \gamma _{F}\right) }=0$. Based on the approval in^14^, a state value represents the probability that an agent can accomplish the specified task starting from this state.

### Reward shaping

The reward function designed above is always zero for any transition between the states $x\notin F_{U}^{\mathcal {{\mathcal {P}}}}$. For instance, given an LDGBA ${\mathcal {A}}_{\phi }$ as shown in Fig. 3, we can obtain its corresponding E-LDGBA $\mathcal {\overline{A}}_{\varphi }$ and construct the EP-MDP ${\mathcal {P}}={\mathcal {M}}\times \mathcal {\overline{A}}_{\phi }$. Then, it can be observed that executing any transition between product states $x=\left( s,l,q_{1},T\right)$ and $x'=\left( s',l',q_{2},T'\right)$ always renders a zero reward for any MDP state $s,s' \in S$. We propose a potential function $\Phi (x):X\rightarrow {\mathbb {R}}$ to increase the reward density and redesign the reward function as below.

13 $$\begin{aligned} R'\left( x,u^{{\mathcal {P}}},x'\right) =R\left( x\right) +\gamma \left( x\right) \cdot \Phi \left( x'\right) -\Phi \left( x\right) \end{aligned}$$

Given an EP-MDP $\mathcal {{P}=M}\times \mathcal {\overline{A}}_{\phi }={\left( X,U^{{\mathcal {P}}},p^{{\mathcal {P}}},x_{0},F^{{\mathcal {P}}},f_{V},T,{\mathcal {B}}\right) }$, there exists a set automaton accepting states, i.e., $\overline{Q}_{F}=\left\{ \overline{q} \in \overline{Q} \vert \overline{q} \in \overline{F}_{i},\forall i\in \left\{ 1,\ldots f\right\} \right\}$. If an agent visits a product state $x=\left( s,l,q_{1},T\right) =(s,l,\overline{q})$ and it is its first time to visit the associated automaton state $\overline{q}$ that belongs to $\overline{Q}\setminus \left( \overline{Q}_{F}\cup \overline{q}_{0}\cup \overline{Q}_{sink}\right)$, the agent will receive a positive reward. Such a modification on the reward design will enhance the guiding of task satisfaction during the learning process via model checking because any automaton state in $\overline{Q}\setminus \left( \overline{Q}_{F}\cup \overline{q}_{0}\cup \overline{Q}_{sink}\right)$, i.e., the one that can reach any accepting set, has to be explored starting from the initial automaton state $\overline{q}_0$.

We design another tracking-frontier set $T_{\Phi }$ to keep tracking unvisited automaton states in $T_{\Phi 0}=\overline{Q}\setminus \left( \overline{Q}_{F}\cup \overline{q}_{0}\cup \overline{Q}_{sink}\right)$. It shall be noted that $T_{\Phi }$ is different from *T*, defined in Section "Embedded LDGBA", which tracks unvisited accepting sets. Initially, $T_{\Phi }$ is set as $T_{\Phi 0}$. Then, it is updated as below at each transition from $\left( s,l,\overline{q}\right)$ to $\left( s',l',\overline{q}'\right)$ after taking action $u^{{\mathcal {P}}}$.

14 $$\begin{aligned} f_{\Phi }\left( \overline{q}',T_{\Phi }\right) =\left\{ \begin{array}{cc} T_{\Phi }\setminus \overline{q}', &{} \text {if }\overline{q}\in T_{\Phi },\\ T_{\Phi 0}\setminus \overline{q}' &{} \text {if }{{\mathcal {B}}}={\text {True}},\\ T_{\Phi }, &{} \text {otherwise. } \end{array}\right. \end{aligned}$$

After ${\mathcal {B}}$ becomes ${\text {True}}$ in $f_{V}$ , i.e., the agent has visited all accepting sets, $T_{\Phi }$ will be reset as $T_{\Phi 0}$. Consequently, the potential function $\Phi \left( x\right)$ at $x=\left( s,l, \overline{q}\right)$ is proposed as:

15 $$\begin{aligned} \Phi \left( x\right) =\left\{ \begin{array}{cc} 1-r_{F}, &{} \text {if }\overline{q}\in T_{\Phi },\\ 0, &{} \text {otherwise} \end{array}\right. \end{aligned}$$

where $r_F$ is the discount factor as defined in Section "Base reward". According to Equation (15), the potential function equals $1-r_{F}$ or 0 for unvisited or visited automaton states. This design will enhance the efficiency of exploration. Based on the shaped reward in Equation (13), the expected return that an agent can collect from state $x\in X$ under policy $\varvec{\pi }$ can be expressed as

16 $$\begin{aligned} U^{\varvec{\pi }}\left( x\right) ={\mathbb {E}}^{\varvec{\pi }}\left[ \sum _{i=0}^{\infty }\gamma ^{i}\left( x_{i}\right) \cdot R'\left( x_{i},u^{{\mathcal {P}}},x_{i+1}\right) \vert x_{0}=x \right] . \end{aligned}$$

#### Theorem 1

Given an EP-MDP $\mathcal {{P}=M}\times \mathcal {\overline{A}}_{\phi }$, by selecting $\gamma _{F}\rightarrow 1^{-}$ and applying a shaped reward function (13), the optimal policy $\varvec{\pi }^{*}$ can maximize the expected return in Equation (16). This policy also maximizes the probability of task satisfaction, i.e., $\Pr ^{\varvec{\pi }^{*}}\left[ x\models \text {Acc}_{{\mathcal {P}}}\right] =\Pr _{max}\left[ x\models \text {Acc}_{{\mathcal {P}}}\right]$.

#### Proof

First, theorems 1-3 of^15^ have verified that by applying the base reward design in Equations (11) and (12), optimizing the expected return can guarantee the optimal policy satisfying the specified LTL task with the maximum probability. Then, the work of^38^ has shown that such an optimal policy remains invariant by applying a shaped reward in Eq. (13). $\square$

To be brief, we proposed a reward-shaping scheme to overcome the issues of sparse reward in an EP-MDP and guarantee that an agent can learn the optimal policy to maximize the probability of task satisfaction.

## Safety value functions

### State and action safety values

Given an MDP ${\mathcal {M}}=\left( S,A,p_{S}, s_{0},\Pi ,L\right)$, the agent is located at the current state $s \in S$. Assuming that the agent can observe the label of its current state only but can record the safety status of the states it has visited as

17 $$\begin{aligned} u_s\left( s\right) =\left\{ \begin{array}{cc} 0 \quad &{} \text {if } L(s)\subseteq L_{us}\\ 1 \quad &{} \text {otherwise} \end{array}\right. \end{aligned}$$

where $L_{us} \subset \Pi$ is a set of unsafe labels, which is a subset of the set of atomic propositions, $\Pi$.

The transition probability $p_S$ is assumed to be unknown to the agent. However, the agent can estimate the transition dynamics based on the observation history. In other words, the agent also records the number of times it executes action *a* at state *s*, i.e., ${\mathcal {N}}(s,a)$, and the number of times it reaches the next state $s'$ after taking *a* at *s*, i.e., $N(s,a,s')$. Consequently, the current belief of the agent about its transition dynamics can be expressed below by considering the Maximum Likelihood Estimation (MLE)^39^ for the mean transition probability function.

18 $$\begin{aligned} \tilde{p}_S(s,a,s') = \frac{N(s,a,s')}{{\mathcal {N}}(s,a)} \end{aligned}$$

A state safety value function $V_s(s)$ is introduced here to represent the minimum probability of the agent moving to a safe state after taking action. It can be estimated via the Bellman update^17,40^:

19 $$\begin{aligned} V_s(s) = \min _{a \in A(s)} \sum _{s'} \tilde{p}_S(s,a,s') u_s(s'). \end{aligned}$$

Then, here defines an action safety value function, representing the maximum probability of the agent staying safe after taking action *a* at state *s*, as

20 $$\begin{aligned} Q_s(s,a) = \sum _{s'} \tilde{p}_S(s,a,s') V_s(s') . \end{aligned}$$

If considering environment uncertainty, a labeling probability function exists such that a state may be labeled “unsafe” with a probability. Consequently, Equation (17) needs to be rewritten as below to update the state’s safety status at every visit.

21 $$\begin{aligned} u_s\left( s\right) =\left\{ \begin{array}{cc} u_s(s) - \frac{u_s(s)}{ N_s(s) + 1 } \quad &{} \text {if } L(s)\subseteq L_{us}\\ u_s(s) + \frac{1.0 - u_s(s)}{ N_s(s) + 1 } \quad &{} \text {otherwise} \end{array}\right. \end{aligned}$$

where $N_s(s)$ is the total number of times the agent has visited state *s*, and it needs to be updated afterward as $N_s(s) \leftarrow N_s(s) + 1$.

In addition, it shall be noted that the safety value functions can be calculated in a continuous state space by revising Equations (19) and (20) as

22 $$\begin{aligned} V_s(s) = \min _{a\in A(s)} \frac{1}{\Omega } \int _{\Omega } \overline{P}_S(s,a,s') u_s(s')ds' \end{aligned}$$

and

23 $$\begin{aligned} Q_s(s,a) = \frac{1}{\Omega } \int _{\Omega } \overline{P}_S(s,a,s') V_s(s')ds' \end{aligned}$$

where $\overline{P}_S(s,a,s')$ can be predicted by an artificial neural network in addition to Q-networks if deep Q-learning^41^ is used. Similar to Q-networks, this transition probability network can be trained and updated by a collection of experiences. In this paper, we consider discrete state spaces only.

### Safe reinforcement learning

Similar to Equation (6), in a Q-learning^33^ on an EP-MDP ${\mathcal {P}}=\left( X,U^{{\mathcal {P}}},p^{{\mathcal {P}}},x_{0},F^{{\mathcal {P}}}, f_{V},T, {\mathcal {B}}\right)$, Q values can be updated below after the agent takes action $u^{{\mathcal {P}}}$ and moves from one state (*x*) to another state ($x'$).

24 $$\begin{aligned} \begin{aligned}Q\left( x,u^{{\mathcal {P}}}\right) {{\leftarrow }}&\left( 1-\alpha \right) Q\left( x,u^{{\mathcal {P}}}\right) \\&+\alpha \left[ R'\left( x,u^{{\mathcal {P}}}, x'\right) +\gamma \left( x\right) \cdot \underset{\overline{u}^{{{\mathcal {P}}}}\in U^{{\mathcal {P}}}}{\max }Q\left( x',\overline{u}^{{\mathcal {P}}}\right) \right] \end{aligned} \end{aligned}$$

The action safety value function in (20) can be incorporated with the Q-value for safe learning. It shall be noted that $\epsilon$-transitions don’t influence the generated policy. Therefore, the action selection technique during the learning process in an EP-MDP ${\mathcal {P}}$ is proposed as:

- if $\{ \epsilon \} \subset U^{\mathcal {P}}(x)$ where $x=(s,l,q,T)$, select action $u^{\mathcal {P}} \in \{ \epsilon \}$.
- if $U^{\mathcal {P}}(x) = A(s)$, section action $u^{\mathcal {P}}(x) \in A(s)$ based on the $\varepsilon$-greedy algorithm as
  25 $$\begin{aligned} u^{\mathcal {P}}\left( x\right) =\left\{ \begin{array}{cc} \underset{a \in A(s)}{\textrm{argmax}} \left[ Q(x,a) + \beta Q_s(s,a) \right] \quad &{} \text {with probability 1-} \varepsilon \\ \text {any action} \left( a \in A(s) \right) \quad &{} \text {with probability } \varepsilon \end{array}\right. \end{aligned}$$
  where $\beta \in [0,1]$ is a parameter bias in selecting safe actions with the importance of action safety.

According to the reward function employed in Equation (11), an action value, i.e., *Q*(*s*, *a*), represents the probability of the agent accomplishing the specified task by taking action *a* at state *s*. Since both represent probabilities, it is natural to combine the action value function and the action safety value function linearly for decision-making in Equation (25). For other definitions of reward functions, Equation (25) might need to be revised^17^.

The safe RL algorithm is described in Algorithm 1, and the product states are generated on the fly based on Definition 4. It shall be noted that the action safety values in Equation (20) can be globally evaluated at the beginning of each learning episode (using collecting data as prior knowledge) as shown in Algorithm 1 or locally updated at the current state and its neighboring states at each step. Our simulations showed that either approach could dramatically reduce the times the agent encounters unsafe states.
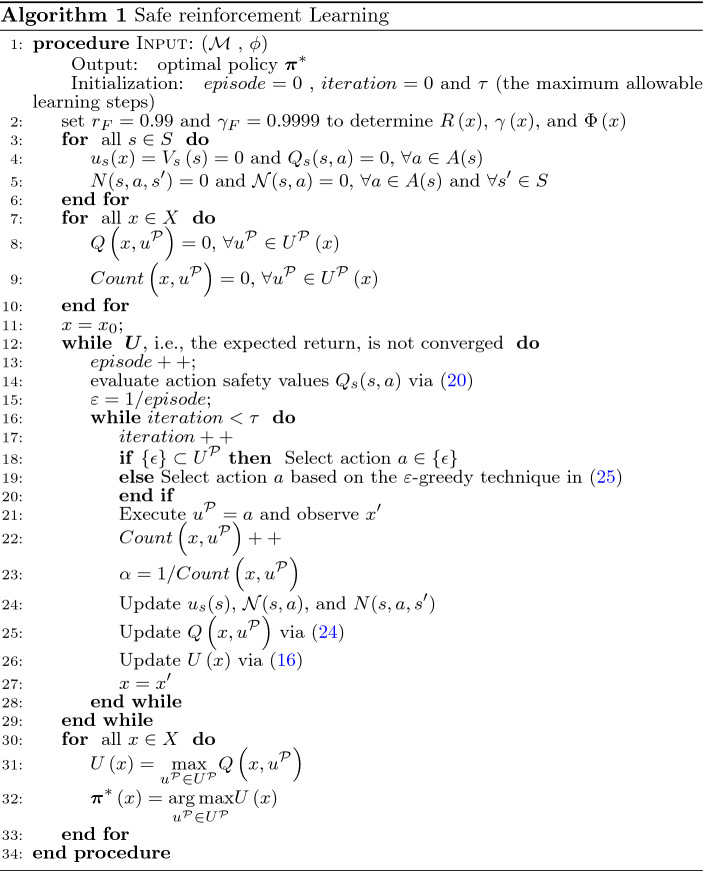


## Quantum action selection

### Grover’s algorithm

Grover’s algorithm^42^, sometimes referred to as Grover Search, quantum search, or quantum database search, is a quantum algorithm for searching through a non-ordered list. It can be used to invert a function, to which there are many possible solutions, but only one is correct. Grover’s algorithm can find the solution out of the pack much faster than any classical algorithm. Searching through the database with *N* items using a classical approach requires *O*(*N*) evaluations (on average *N*/2 evaluations). However, Grover’s algorithm can complete such a search using only $O(\sqrt{N})$ evaluations.

The search problem we consider here consists of three steps: marking the desired basic state within the $2^n$ space represented by an n-qubit quantum state, applying Grover’s algorithm on the quantum state, and finding the desired basic state with a single measurement. At first, we prepare an n-qubit quantum state, which is initialized as $\vert q_1 q_2 \dots q_n \rangle = \vert 00 \dots 0 \rangle$, by applying an *H* gate to each qubit,

26 $$\begin{aligned} \vert \psi \rangle = H^{n} \vert 00 \dots 0 \rangle = \frac{1}{\sqrt{2^n}} \sum _{k=0}^{2^{n}-1} \vert k \rangle \end{aligned}$$

which results in an equal superposition of all $2^n$ basic states.

We use an ‘oracle’ operator $U_d$ to mark the desired basic state, e.g., $\vert d \rangle$, in the above superposition $\vert \psi \rangle$, as described in^43^. With the assistance of ancilla qubits, this operator utilizes *X* gates and an *n*th-order *CNOT* gate to flip the sign of the desired state. It shall be noted that the *n*th-order *CNOT* gate uses *n* qubits as the control qubits with the ancilla qubit as the target. Consequently, the n-qubit quantum state becomes

27 $$\begin{aligned} \vert \phi \rangle = U_d \vert \psi \rangle = \frac{1}{\sqrt{2^n}} \sum _{k \ne d} \vert k \rangle - \frac{1}{\sqrt{2^n}} \vert d \rangle \end{aligned}$$

Next, a Grover diffusion operation is conducted to amplify the amplitude of the desired basic state. Mathematically, the Grover diffusion operation is equivalent to a reflection of the average amplitude of all $2^n$ basic states, and it can be expressed as the Grover diffusion operator^43^, $U_s = 2 \vert \psi \rangle \langle \psi \vert - I$, on the n-qubit system, $\vert \phi \rangle$ in Equation (27). However, $\vert \psi \rangle \langle \psi \vert$ is not a unitary operator, so it is not physically realizable. It has been demonstrated that *H* gates and the oracle operator can be utilized to realize the Grover diffusion operator^43^. Theoretically, the optimal number of times to conduct the Grover iteration, consisting of oracle and diffusion operations, is $r=\frac{\pi }{4 \theta } - \frac{1}{2}$, where $\sin {\theta } = \frac{1}{\sqrt{N}}$ and $N=2^n$. When $N>> 1$, it can be approximated as $\frac{\pi \sqrt{N}}{4}$.

The last step is to measure the resulting n-qubit quantum state and find the desired basic state. For a two-qubit system, after one Grover iteration, the quantum state becomes

28 $$\begin{aligned} U_s U_d H^2 \vert 00 \rangle = - \vert d \rangle \end{aligned}$$

Theoretically, the desired basic state $\vert d \rangle$ is surely found. For a three-qubit system, after two Grover iterations, the quantum state becomes

29 $$\begin{aligned} (U_s U_d)^2 H^3 \vert 000 \rangle = -0.08839 \sum _{k \ne d} \vert k \rangle + 0.97227 \vert d \rangle \end{aligned}$$

Therefore, there is a 5.5$\%$ probability of obtaining a wrong basic state other than $\vert d \rangle$.

### Quantum action selection

Here we propose the quantum action selection technique that can replace the $\varepsilon$-greedy action selection from the quantum computing point of view. According to the number of available actions, e.g., *M*, *n* qubits are utilized to form a superposition of $2^n$ basic states, representing discrete actions in an MDP problem. *M* and *n* are characterized by the following inequality:

30 $$\begin{aligned} M \le 2^n < 2M \end{aligned}$$

Taking the example of a mobile robot moving in a grid world, if the robot can move “up”, “down”, “left” and “right”, those actions are represented by two-qubit basic states, $\vert 00 \rangle$, $\vert 10 \rangle$, $\vert 01 \rangle$, and $\vert 11 \rangle$, respectively. After applying the *H* gate on each qubit of the initial quantum state $\vert 00 \rangle$ as in Equation (26), we can obtain an equal superposition of $\frac{1}{2} (\vert 00 \rangle +\vert 10 \rangle +\vert 01 \rangle +\vert 11 \rangle )$, meaning that each action will have the same probability of being selected if we measure this two-qubit state once. To select the best action, the Grover algorithm needs to be applied.

Based on the combination of action values and action safety values, as shown in Equation (25), the basic state corresponding to the action associated with the highest value is marked by the oracle function in Equation (27). Then, the amplitude of this basic state is amplified via the Grover diffusion operation, so the probability of selecting the best action is higher than the others when measuring the quantum state. However, as demonstrated in the previous subsection, after one Grover’s iteration on the equal superposition state $\frac{1}{2} (\vert 00 \rangle +\vert 10 \rangle +\vert 01 \rangle +\vert 11 \rangle )$, it is reduced to the marked basic state with an amplitude of $-1$. In other words, only the best action is selected when measuring this quantum state, as shown in Equation (28). This is equivalent to the greedy action selection.

In another case, a three-qubit system is needed if the robot can conduct eight actions. After applying the quantum action selection technique described above, the marked action always has a high probability, 94.5$\%$, of being selected. Consequently, the balance of exploration and exploitation is not achieved, especially at the beginning of learning.

**Figure 4:**
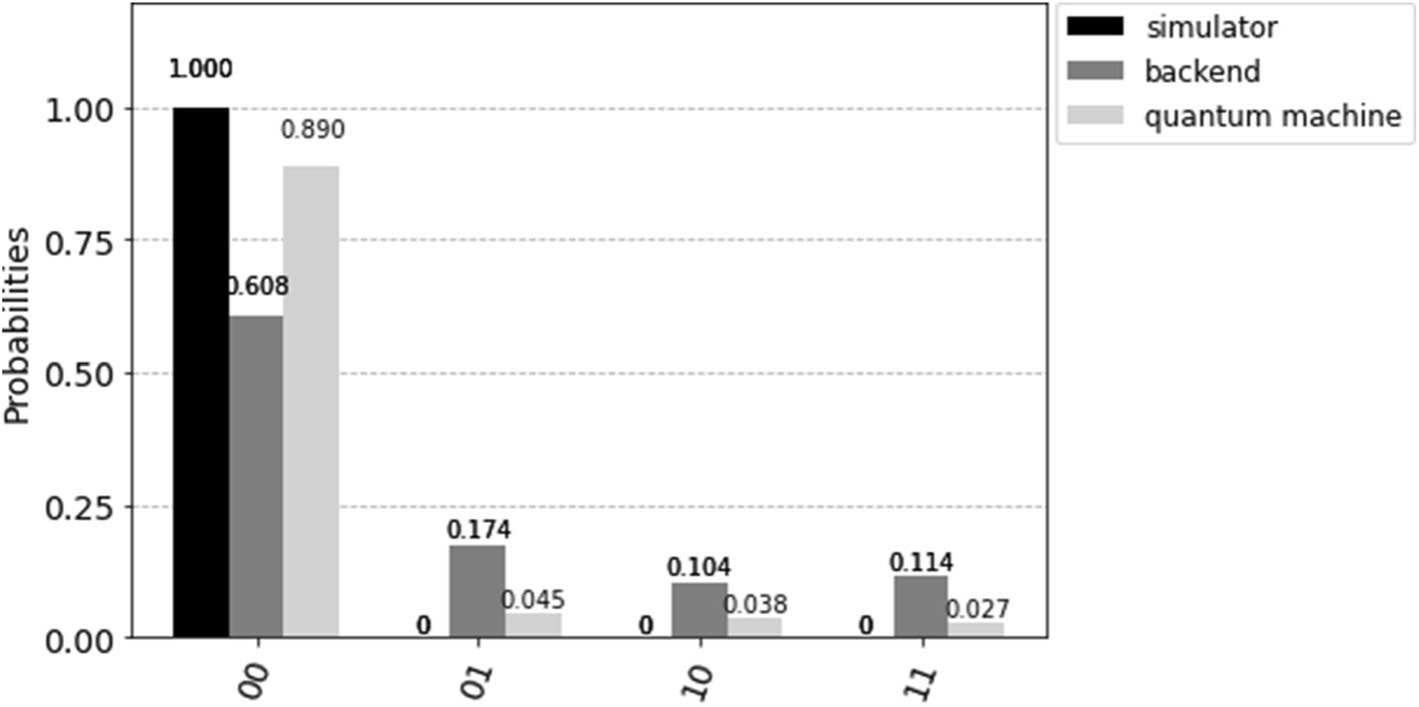
The measurement probability of $-1\vert 00 \rangle$ by using 1) a quantum simulator on a classical computer, 2) a quantum machine as the backend on a classical computer, and 3) a quantum machine.

It shall be noted that the above discussions are theoretical and only validated on the quantum simulators on classical computers. Indeed, quantum computers always have decoherence and noise issues, so it would be impossible to follow the exact probabilities when measuring the quantum state. Continuing the above example of a 2-qubit system, if the best action is “up”, the basic state $\vert 00 \rangle$ is marked. After one Grover iteration, the resulting quantum action state is $-\vert 00 \rangle$, and it is then measured with 1024 shots. We executed the simulation in three different ways: 1) using a quantum simulator of qiskit^44^, an open-source software development kit (SDK), on a classical computer, 2) using ibmq$\_$Belem (an IBM 5-qubit quantum machine) as the backend on a classical computer, and 3) using ibmq$\_$Belem through the IBM quantum composer online. The calculated probabilities are shown in Fig. 4.

It can be seen that the quantum simulator (on a classical computer) doesn’t suffer from different error sources because those errors can be corrected with a small amount of extra storage and logic in the classical computer. Larger errors are induced when using a quantum machine as the backend than when directly using a quantum machine. Although some quantum error correction algorithms exist, they utilize many qubits, and only a few qubits are left for actual quantum computation. Therefore, due to the limited number of qubits on the existing quantum computers, no hardware platform can currently conduct robust error corrections for large-scale quantum computation. As mentioned in^45^, quantum technologists are putting their efforts into more accurate quantum gates and, eventually, fully error-tolerant quantum computing.

**Figure 5:**
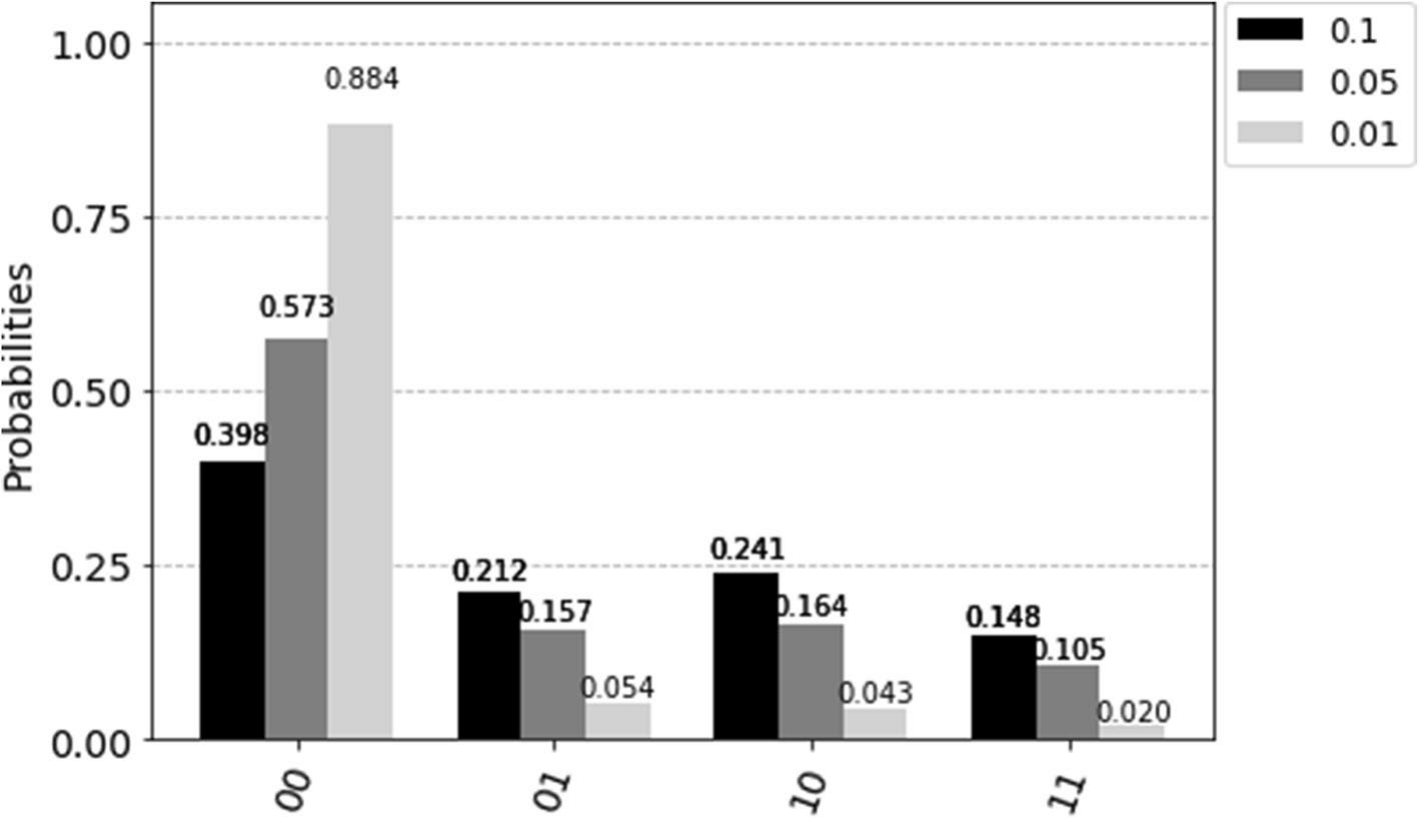
The measurement probability with three different noise levels.

However, in our quantum action selection method, we take advantage of gate noises and measurement errors to balance the exploration and exploitation when using the quantum simulator on a classical computer. We employ the noise model from qiskit to implement the depolarizing and measurement noise. The first noise model results in an imperfection in quantum gate operations, i.e., replacing the state of any qubit with a completely random state with a probability $p_{gate}$. Our quantum action selection method applies this noise model on *H*, *X*, and *CNOT* gates. The other noise model flips between $\vert 0 \rangle$ and $\vert 1 \rangle$ immediately before measurement with probability $p_{meas}$. It shall be noted that $p_{gate}$ and $p_{meas}$ are heuristic parameters, and it is recommended that the values are initialized as $p_{gate}=p_{meas}=0.1$ and then reduced to 0.01 for 2-qubit quantum states in our simulations. The probabilities of action selection, corresponding to the example in Fig. 4 at different noise levels, are shown in Fig. 5.

The flowchart of the quantum action selection method is summarized below. We implement the developed quantum action selection technique in our safe RL method (Algorithm 1), resulting in a Quantum Safe Q-learning (QSQ-learning) method. Algorithm 2 can be implemented in any other RL algorithm to replace $\varepsilon$-greedy for action selection.
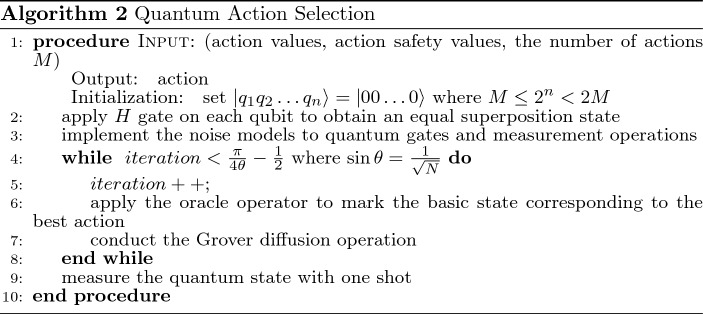


## Simulations and discussions

We study two examples to apply the developed QSQ-learning algorithm for motion planning in grid worlds in this study. The noise in the quantum simulator is set as 0.1 and then slowly decreased to 0.01 for action selection. The reward calculation uses $\gamma _B=0.99$ and $\gamma =0.99999$ in Eq. (11).

### Motion planning with safe absorbing states

**Figure 6:**
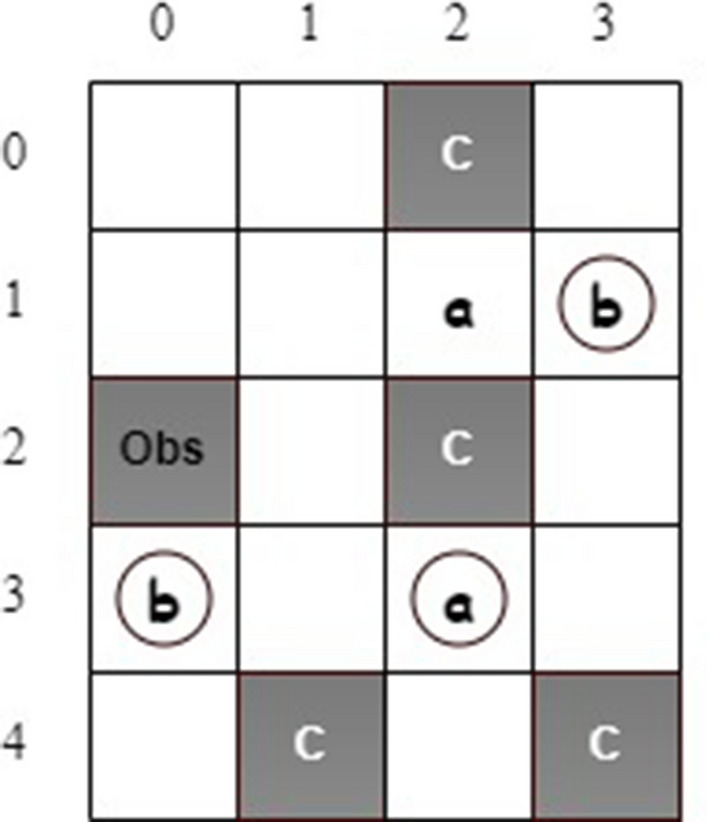
The grid-world with safe absorbing states.

Figure 6 shows a grid world with safe absorbing states^13^. A mobile robot can take four actions: *left*, *up*, *right*, and *down*. The robot receives a reward if it reaches states labeled *a* or *b*. Also, three states, with circles, are safe absorbing states. Once the robot reaches one of them, it will stay in this state no matter which action is taken. States labeled *c* are unsafe, and “Obs” represents an obstacle. In this example, the objective of motion planning is finding a policy so that the robot can eventually always visit a safe absorbing state while avoiding unsafe states. This task can be specified as the below LTL formula.

31 $$\begin{aligned} \varphi _1 = \left( \lozenge \square a \vee \lozenge \square b \right) \wedge \square \lnot c \end{aligned}$$

**Figure 7:**
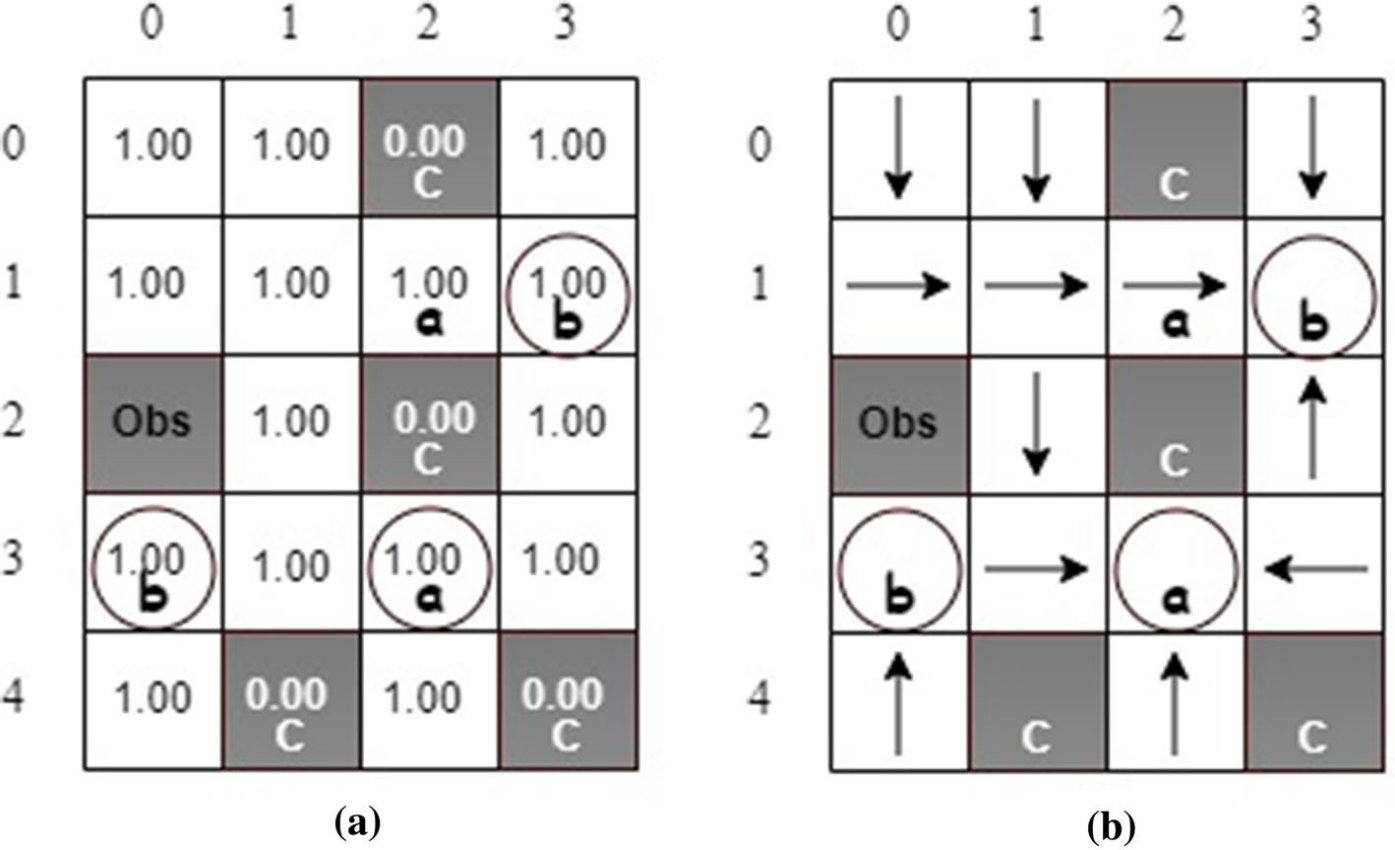
The schemes of (**a**) state values and (**b**) an optimal policy.

It shall be noted that state (1, 2) (labeled *a*) is not a safe absorbing state. Therefore, although the robot receives a reward for visiting this state, it moves away after taking any action. We first consider deterministic actions, i.e., there is only a single next state when the robot takes action at the current state. We conducted five simulations with safety value functions and another five without safety value functions and obtained the same optimal policies. Figure 7 illustrates state values and an optimal policy. It can be seen that all states, except unsafe and obstacle states, ensure the 100$\%$ probability of satisfying the task if the robot starts from one of those states and follows the optimal policy. It is worth mentioning that there is more than one optimal policy for motion planning in this example.

We conduct the simulations with and without safety value functions for comparison. Each simulation takes 10,000 episodes with 200 steps per episode. We investigate the number of times the robot visits unsafe states while learning optimal policies. It is found that implementing safety value functions could dramatically reduce the number of times to visit unsafe states. Specifically, without using safety value functions, the robot visits unsafe states an average of 950 times, while it visits unsafe states only 177 times when considering both safety values and Q-values in decision-making.

**Figure 8:**
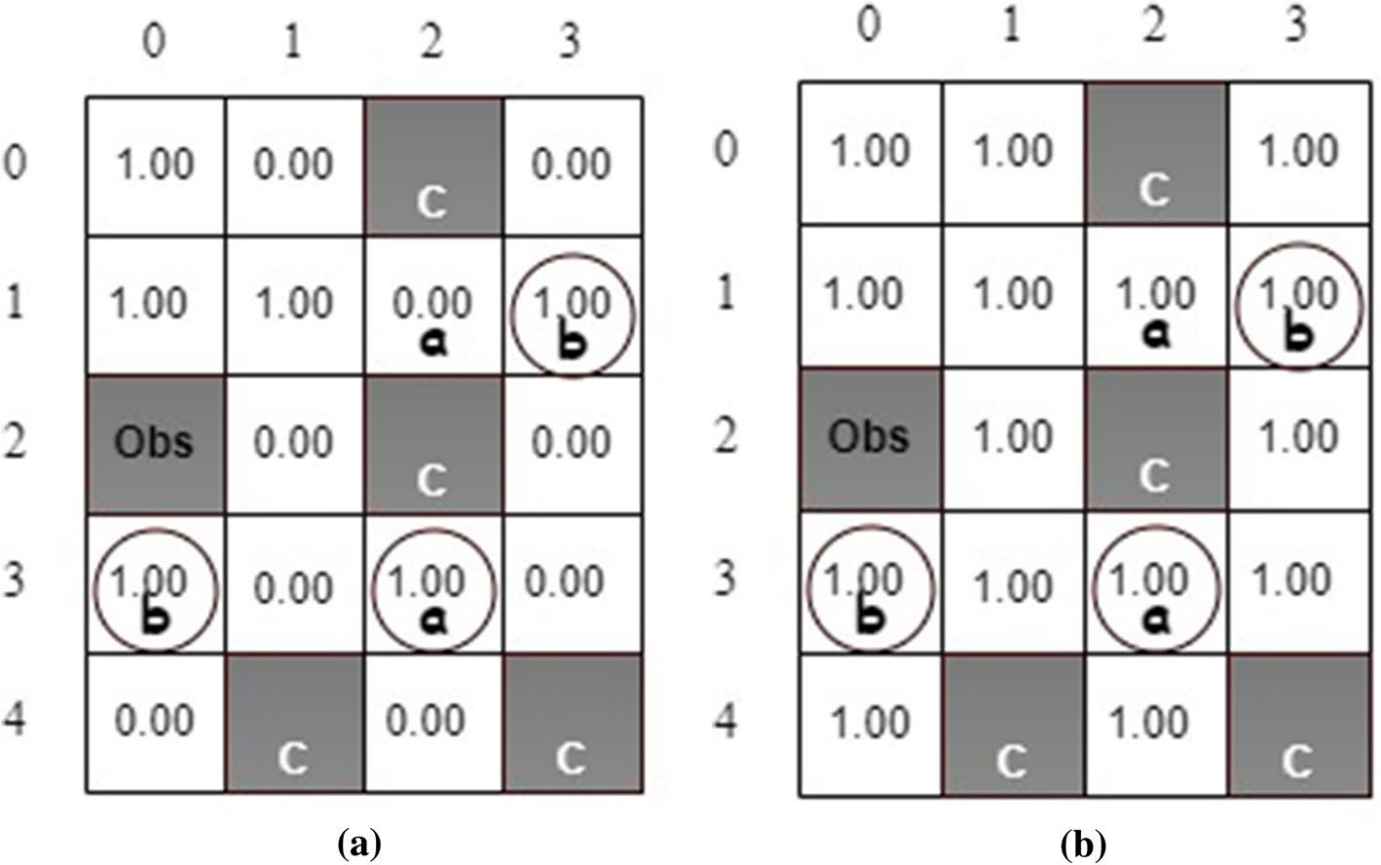
The results of (a) state safety values and (**b**) the maximum action safety values.

The state safety values and the maximum action safety values are shown in Fig. 8. The safety state value represents the minimum probability of transitioning the agent from the current state to a safe state. For example, the robot can move from state (0, 1) to unsafe state (0,2) by taking action *right* so that the state safety value is 0 at state (0, 1). However, after taking action *down*, the robot can move from state (0, 1) to state (1, 1), which is a safe state. Consequently, the maximum action safety value is 1.00 at state (0, 1) as shown in Fig. 8(b).

**Figure 9:**
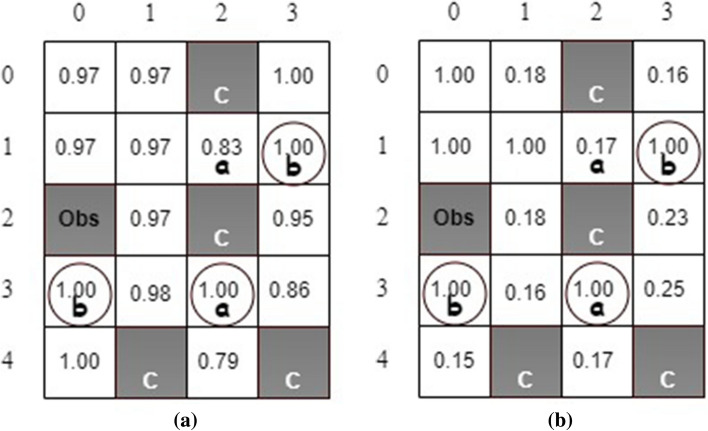
The estimated (**a**) state values and (**b**) state safety values for stochastic actions.

We also consider the scenario with action uncertainties due to the actuator malfunction. Therefore, the considered MDP is stochastic. After an action is selected, the robot moves in the desired direction with a probability of $80\%$. On the other hand, it can go in each side direction with a probability of $10\%$. Figure 9 illustrates the estimated state values and state safety values after one simulation via the developed QSQ-learning. Theoretically, the state values, i.e., the maximal task-satisfaction probabilities, shall be 1.0, 0.9, or 0.8 at all non-unsafe states due to the action uncertainties. For example, the robot can take a *right* action at state (1, 2) to a safe absorbing state, (1, 3). Therefore, the probability of the robot fulfilling the task requirement is $80\%$. The estimated state values in Fig. 9a from our simulation agree with the theoretical predictions.

**Figure 10:**
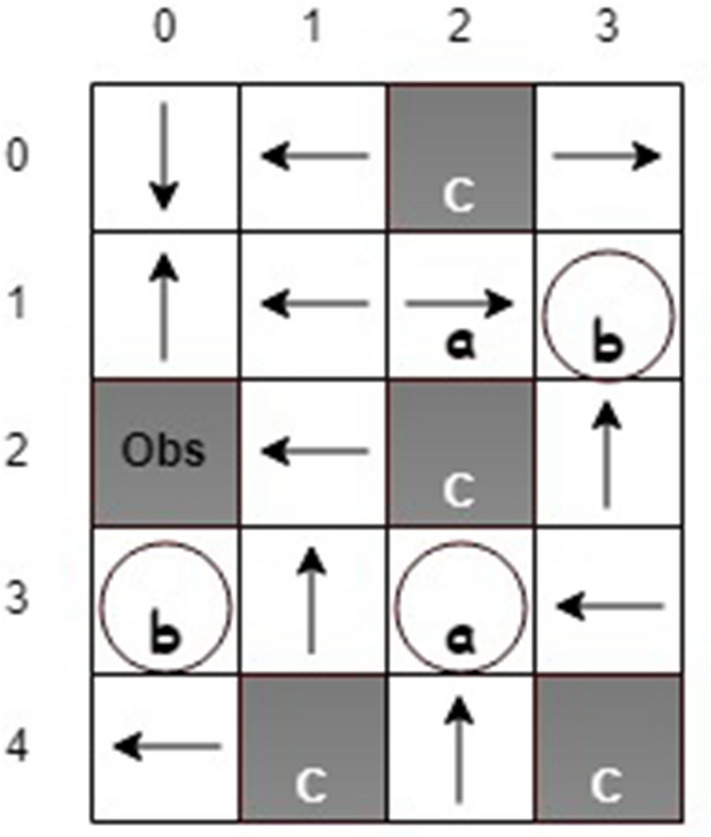
The generate optimal policy.

**Figure 11:**
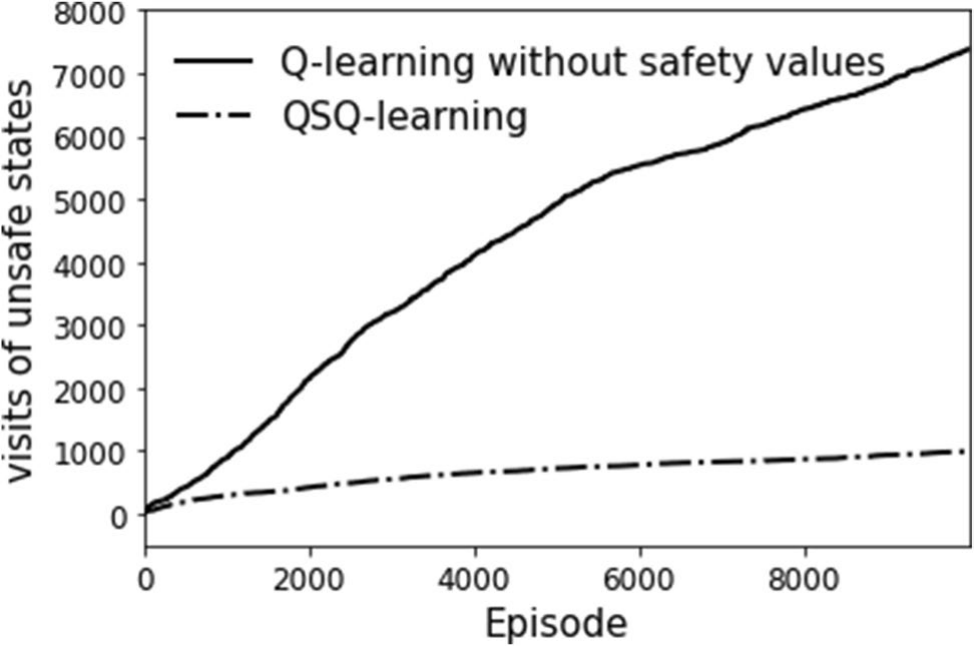
The evolution of visiting unsafe states.

On the other hand, the state safety value at state (1, 2) in Fig. 9b is expected to be 0.2 because the robot has a minimum probability of $20\%$ of reaching a safe state after taking action *up* or *down* (the estimated safety value at this state is 0.17). One optimal policy is obtained, as shown in Fig. 10. The state values indicate at least one path generated from the optimal policy so that the robot can accomplish the task. We investigate the evolution of the times the robot visits unsafe states during the learning and compare the results with the one from Q-learning without safety value functions, as shown in Fig. 11. It can be seen that implementing safety value functions dramatically reduces the number of visits to unsafe states up to $87\%$.

Unlike a previous work^17^, in which an agent was able to observe the safety statuses of its neighboring states, it is assumed that the agent can observe the current state’s safety status only. Therefore, the agent must visit unsafe states due to exploration, especially at the beginning of learning, to obtain enough information for calculating the safety value functions. However, considering safety in action selection significantly saves the number of visits to unsafe states compared to the conventional RL methods.

### Slippery grid-world

**Figure 12:**
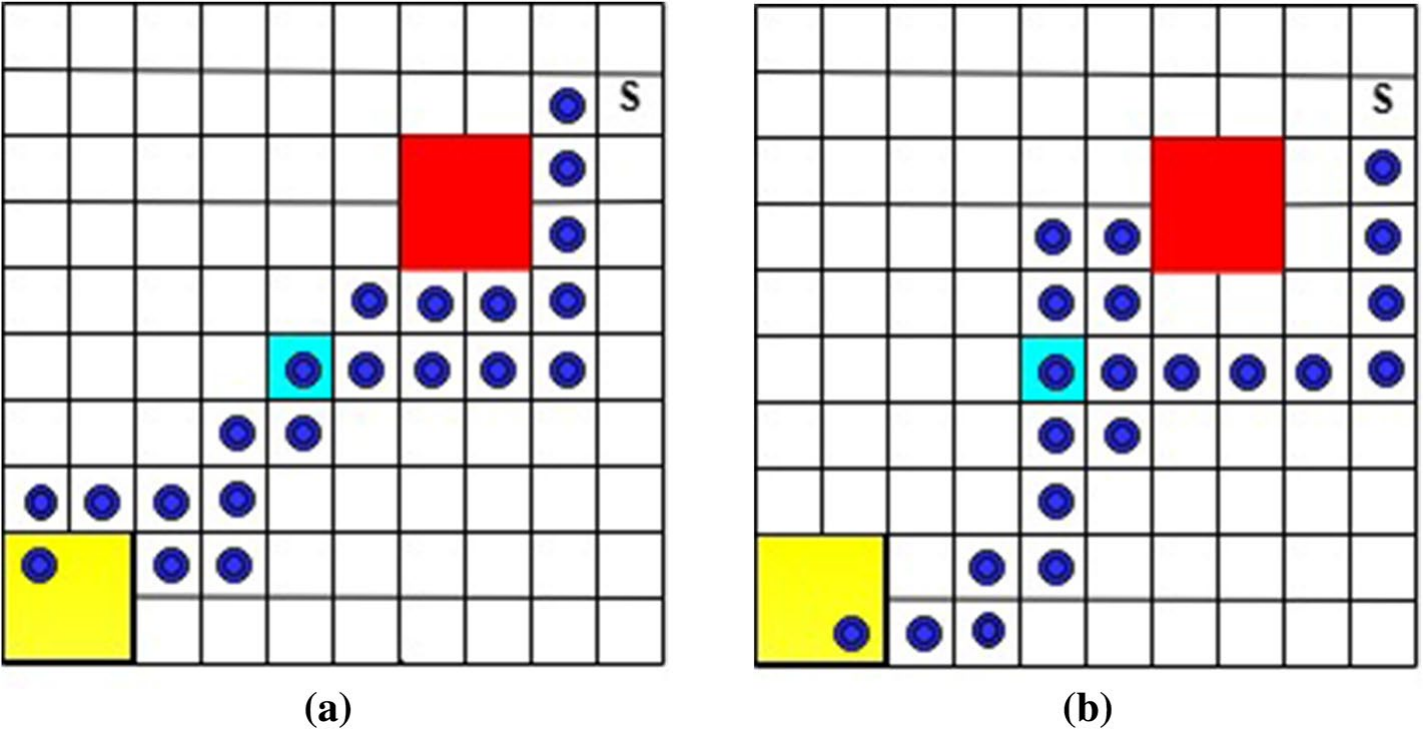
The generated trajectories from the optimal policy learned via Q-learning without safety value functions.

In this example, a robot moves on a $10 \times 10$ grid world, shown in Fig. 12, where the robot can “slip” to any adjacent state with a probability of $15\%$ when taking action. The robot takes off from the initial state (marked as “S” in Fig. 12) and tries to visit *goal*1 (cyan) and then *goal*2 (yellow) for infinite times while avoiding unsafe states (red). The task can be specified as the following LTL formula.

32 $$\begin{aligned} \varphi _2 = \square \left( goal1 \; {\mathcal {U}} \; goal2 \right) \wedge \square \lnot \; unsafe \end{aligned}$$

**Figure 13:**
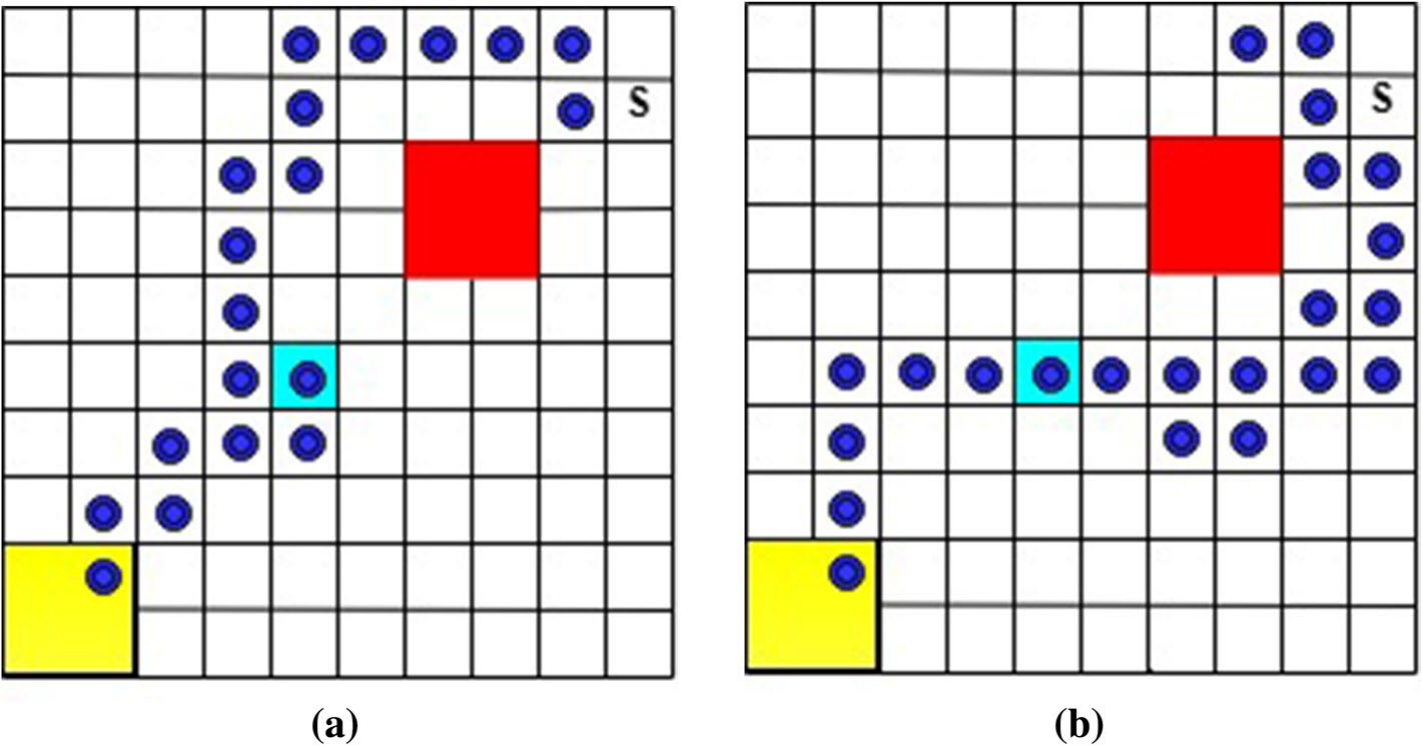
The generated trajectories from the optimal policy learned via QSQ-learning.

We conducted 10 simulations for both Q-learning (without safety value functions) and the developed QSQ-learning. Each simulation consists of 1000 episodes with 4000 steps per episode. Two trajectories, induced from the optimal policies via Q-learning, for the first round of visiting *goal*1 and then *goal*2 are illustrated in Fig. 12. In addition, Fig. 13 includes two trajectories generated from the optimal policies learned via QSQ-learning. For each learning method, we record the maximum and minimum times the robot visits unsafe states during the simulations and list them in Table 1. It can be seen that the robot visits unsafe states many fewer times when implementing unsafe value functions in learning.

**Table 1:** The number of times the robot visits unsafe states during learning.

	maximum times	minimum times
Q-learning without safety values	160,937	157,192
QSQ-learning	2,294	962

## Conclusions and future work

This paper presents safe reinforcement learning to find RL control policies that satisfy LTL specifications over finite and infinite horizons. The developed reward-shaping process further improves the reward density and guides the LTL satisfaction with maximum probability, while the safe padding technique utilizes the properties of E-LDGBA and maintains the safe exploration without influencing the original probabilistic guarantee. In addition, the quantum action selection technique provides an alternative approach to balancing exploration and exploitation during RL while taking advantage of quantum computing.

The state labels, including safety labels, are atomic propositions. In real-world problems, the agent can acquire the labels based on the collected information via perception sensors and its prior knowledge base. The safety value function proposed in this paper represents the probability of safely reaching the next state after taking a selected action. Such a concept can be extended to calculate the expected probability of reaching safe states after taking action and following the current policy within a finite or infinite horizon. In addition, the idea of calculating safety value functions for a continuous state space, proposed as Equations (22) and (23), will be refined in future studies.

Quantum computing is powerful because the quantum algorithms, such as Grover’s search algorithm, have lower computational complexities than their classical equivalents. However, current quantum computers are relatively small (up to 433 qubits in the largest quantum computer built by IBM) and noisy (not fault-tolerant). Indeed, we are in the era of Noisy Intermediate-Scale Quantum (NISQ)^45^. Consequently, Variational Quantum-Classical (VQC) algorithms have become popular in deploying quantum algorithms on near-term quantum devices. In VQC algorithms, classical computers perform the overall computation task on information they acquire from running calculations on a quantum computer. Our quantum safe RL approach adopts the same strategy: the learning process is conducted on a classical computer while the action is selected via quantum computing. The proposed quantum action selection algorithm results in parameterized quantum circuits, which are relatively small, short-lived, and thus suitable for NISQ computers. Although quantum action selection is performed on the quantum simulator in our simulations, it can be run on a quantum machine as the backend (see Fig. 4).

Although quantum computing theoretically promises to be exponentially faster than classical computers, we don’t think the computation would be faster for our simulation examples, even using a quantum machine to conduct action selection because the action spaces are small. However, we expect our method to take advantage of quantum physics’s fundamental properties, especially superposition when handling problems with large/continuous action spaces. For example, our previous work^1^ studied motion planning for Mars exploration cases with a large-scale continuous environment. Our method can be utilized to solve the same problems by applying a fine-scale discretization to the action space since an *n*-qubit system can represent $2^n$ actions. The method can be extended to the discretization of a continuous state space with quantum states.

In addition, the proposed quantum action selection method can also be applied to policy-based RL methods, in which the actions are predicted with various probabilities. In this case, the basic quantum state corresponding to the action with the highest predicted probability would be marked. Considering the above-mentioned motivations and challenges, further research is needed to extend this method to problems with continuous state and action spaces.

## Data Availability

The datasets generated during and/or analysed during the current study are available from the corresponding author on reasonable request.
